# An open-source smartphone app for the quantitative evaluation of thin-layer chromatographic analyses in medicine quality screening

**DOI:** 10.1038/s41598-022-17527-y

**Published:** 2022-08-04

**Authors:** Cathrin Hauk, Mark Boss, Julia Gabel, Simon Schäfermann, Hendrik P. A. Lensch, Lutz Heide

**Affiliations:** 1 Pharmaceutical Institute, Eberhard Karls University Tübingen, Tübingen, Germany; 2 Computer Graphics, Department of Computer Science, Eberhard Karls University Tübingen, Tübingen, Germany

**Keywords:** Analytical chemistry, Computer science, Software, Drug safety, Screening

## Abstract

Substandard and falsified medicines present a serious threat to public health. Simple, low-cost screening tools are important in the identification of such products in low- and middle-income countries. In the present study, a smartphone-based imaging software was developed for the quantification of thin-layer chromatographic (TLC) analyses. A performance evaluation of this tool in the TLC analysis of 14 active pharmaceutical ingredients according to the procedures of the Global Pharma Health Fund (GPHF) Minilab was carried out, following international guidelines and assessing accuracy, repeatability, intermediate precision, specificity, linearity, range and robustness of the method. Relative standard deviations of 2.79% and 4.46% between individual measurements were observed in the assessments of repeatability and intermediate precision, respectively. Small deliberate variations of the conditions hardly affected the results. A locally producible wooden box was designed which ensures TLC photography under standardized conditions and shielding from ambient light. Photography and image analysis were carried out with a low-cost Android-based smartphone. The app allows to share TLC photos and quantification results using messaging apps, e-mail, cable or Bluetooth connections, or to upload them to a cloud. The app is available free of charge as General Public License (GPL) open-source software, and interested individuals or organizations are welcome to use and/or to further improve this software.

## Introduction

An urgent public health challenge of our time is the world-wide spread of substandard and falsified medicines. WHO estimates that 10.5% of the medicines in low- and middle-income countries (LMICs) are substandard or falsified (SF)^1^. SF medicines frequently fail to cure diseases, may cause toxic effects from incorrect active ingredients (APIs) or contaminants, contribute to the spread of anti-microbial resistance, and lead to a loss of confidence of the patients in health services. They also have detrimental economic and socioeconomic impacts^1^. At the same time, the trade with SF medicines is highly profitable for criminals and criminal organisations, with little risk of detection and prosecution^2^.

Pharmacopeial methods for medicine quality analysis and for detection of SF medicines require sophisticated and expensive techniques as well as highly trained personnel, and are therefore difficult to implement in resource-limited settings^3^. Simple and inexpensive field detection devices allow for rapid screening for SF medicines and are therefore useful especially in countries with limited technical capacities^4–6^. The use of screening tools reduces the time between collection of medicine samples and availability of analytical results, helping to prevent that SF medicines reach the patients^7–9^. Medicine quality screening tools based on different technologies have been developed^3,8,10–15^, and reviews of these tools have been published in the last years^4,7,9,16–20^. Recently, a guideline was introduced in the Unites States Pharmacopeia (USP) 42 for the characterization and validation of such screening tools^21^. The United States Pharmacopeial Convention also established a Technology Review Program in which they published evaluations of six medicine screening technologies^22^. The group of Newton recently published a comparative evaluation of 12 screening devices^23–27^. These authors concluded that the evaluation of medicine quality screening devices is still in its infancy, and they emphasized the need for further research^23^.

A key finding of the above-mentioned comparative evaluation was that none of the tested devices was able to accurately identify medicines which contain the declared API in incorrect quantities. The present study attempts to address this gap by introducing a simple, low-cost screening method for the quantification of APIs in medicine quality analysis using the Global Pharma Health Fund (GPHF) Minilab.

The GPHF Minilab is currently the most widely used low-cost screening technology for medicine quality in low-resource settings^28–30^. Out of 48,218 medicine analyses included in an authoritative WHO review^1^, 20,010 had been carried out using the GPHF Minilab. The Minilab relies especially on thin-layer chromatography (TLC), a well-established, low-cost chromatographic technique^30,31^. The current GPHF Minilab manual includes procedures for the TLC analysis of 107 APIs and their common fixed combinations^30^. The quantitative evaluation of the TLC results is based on a visual comparison of the spots of the analysed sample to spots of an authentic reference which correspond to 100% and 80% of the declared amount of the API, respectively. This requires visual inspection skills, and the accuracy of the assessment can be improved by appropriate training^16,32–34^.

The GPHF Minilab reliably confirms the presence or absence of an API. However, it has a limited ability for the detection of products that contain incorrect amounts of the API^3,6,10,13,34,35^. E.g. in a study in the DR Congo and Cameroon, analysis with the GPHF Minilab correctly identified all samples that did not contain the stated API; however, out of 14 extremely substandard samples that contained less than 80% of the declared API, only six (43%) were correctly identified as out-of-specification (OOS)^36^. In the comparative laboratory evaluation of screening devices by the group of Newton^27^, analysis with the GPHF Minilab correctly detected the presence or absence of the declared API in all 77 investigated samples (including 53 samples which contained no API, or the wrong API). Furthermore, Minilab analysis correctly identified 20 (95%) out of 21 samples of simulated medicines which contained only 50% of the declared amount of the API as non-compliant. However, out of another 21 samples which contained 80% of the declared amount of the API, only 5 (24%) were identified as non-compliant.

Automated high performance thin-layer chromatography (HPTLC) in combination with benchtop densitometers have made planar chromatography a powerful tool for the quantification of pharmaceutical products, although the cost of the equipment is quite high^16,37–39^. However, quantification of analytical results from TLC plates is not limited to applications requiring expensive, non-portable benchtop plate readers^40^. Yu et al. designed a simple field detection device for medicine quality screening, taking images of UV-illuminated TLC plates in a 3D printed cradle using a smartphone camera^41^. By analysing the intensity of the TLC spots with a smartphone-based algorithm, they reported to be able to discriminate 5% differences in the content of three selected APIs, i.e. paracetamol, amodiaquine and nevirapine. No detailed validation of the accuracy and precision of this tool was presented, and the imaging software has not been made available to the public, neither commercially nor as open-source software.

Boulgakov et al.^42^ developed a TLC imaging system to quantitively follow the conversion rates in certain chemical reactions. The average error of the determinations was reported to range from 0 to 33%, with higher errors related to incomplete separation in TLC analysis. Recently, the quantitative determination of the antibiotics ofloxacin and ornidazole by TLC after staining with iodine vapour was reported, using a smartphone camera and a freely available smartphone app for quantification^43^. The authors reported to achieve relative standard deviations below 1%, although such a high precision appears surprising also in view of the rapid decay of colour intensity after iodine staining. Tosato et al.^44^ developed a combination of TLC and smartphone-based digital image analysis for the quantification of cocaine and its common diluent phenacetin in seized street drugs, and reported relative standard deviations of 6% and 7% for the quantification of cocaine and phenacetin, respectively. Smartphone-based instrumentations have also been developed for the assessment of multiple analytes^45^.

In the present study, we developed and tested a new Android-based open-source algorithm that can quantify different APIs from smartphone camera images taken of TLC plates prepared according to the GPHF Minilab methods. We carried out a laboratory performance evaluation of this tool in the quantitative analysis of 14 selected essential medicines. The evaluation followed the recent guideline of the USP on the evaluation of screening technologies for assessing medicine quality^21^ and the guideline Q2 (R1) of the International Council for Harmonisation of Technical Requirements for Pharmaceuticals for Human Use (ICH) on validation of analytical procedures^46^, and we assessed accuracy, repeatability, intermediate precision, specificity, linearity, range and robustness of this method.

## Results

### Design of a locally producible box for photographing TLC plates under UV illumination

**Figure 1:**
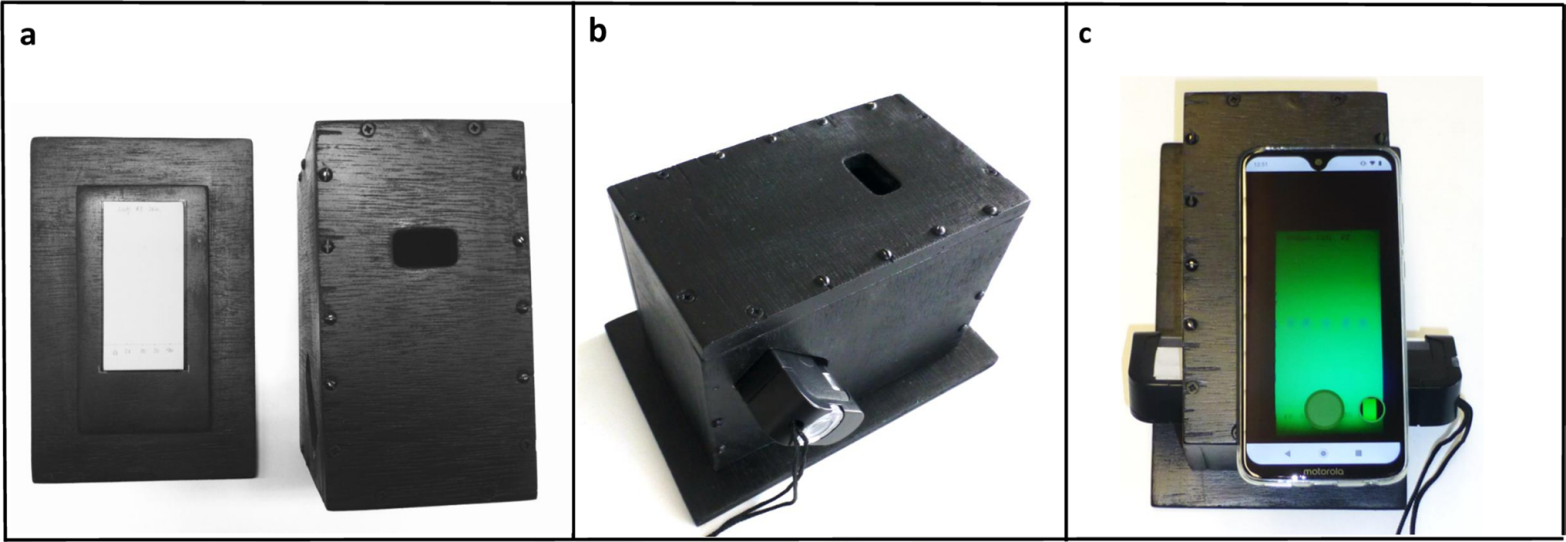
Box for photography of TLC plates under UV illumination. (**a**) Left: Bottom plate with TLC plate. Right: box-shaped lid with openings for the UV lamp and for photography. (**b**) Assembled box with the UV lamp inserted. (**c**) Assembled box with TLC plate, UV lamp, and smartphone.

In TLC analysis with the GPHF Minilab, the spots of the APIs are most commonly visualized by their fluorescence quenching under UV illumination. For photography of such TLC plates under standardized conditions, the 3D-printed cradle described by Yu et al.^41^ was modified here with three aims: (1) creating a design which is locally producible in low-resource settings; (2) better protection of the UV-illuminated TLC plate from ambient light, to improve image quality; (3) usability with smartphones of different sizes. Several designs were developed and tested, resulting in the one depicted in Fig. 1. The wooden box is painted in matte black colour to minimize reflections. It consists of a bottom plate which accommodates the TLC plate in a marked rectangle, and of a box-shaped lid. The lid has openings in the sides for insertion of the battery-operated UV lamp supplied with the GPHF Minilab. A third opening located in the flat upper side enables capturing the TLC plate with any rear-facing smartphone camera. Precise drawings for the construction of the box are given in Supplementary Fig. S2. The box was produced in a workshop of the University of Tübingen, and was subsequently reproduced by a commercial workshop in Germany (see “Methods”), and by a carpenter in Zimbabwe (Supplementary Fig. S5). We found this box to offer easier handling, better protection from ambient light, and cheaper production than the 3D-printed cradle described by Yu et al.^41^.

### Development of a smartphone-based image processing algorithm for quantitative evaluation of TLC analysis

**Figure 2:**
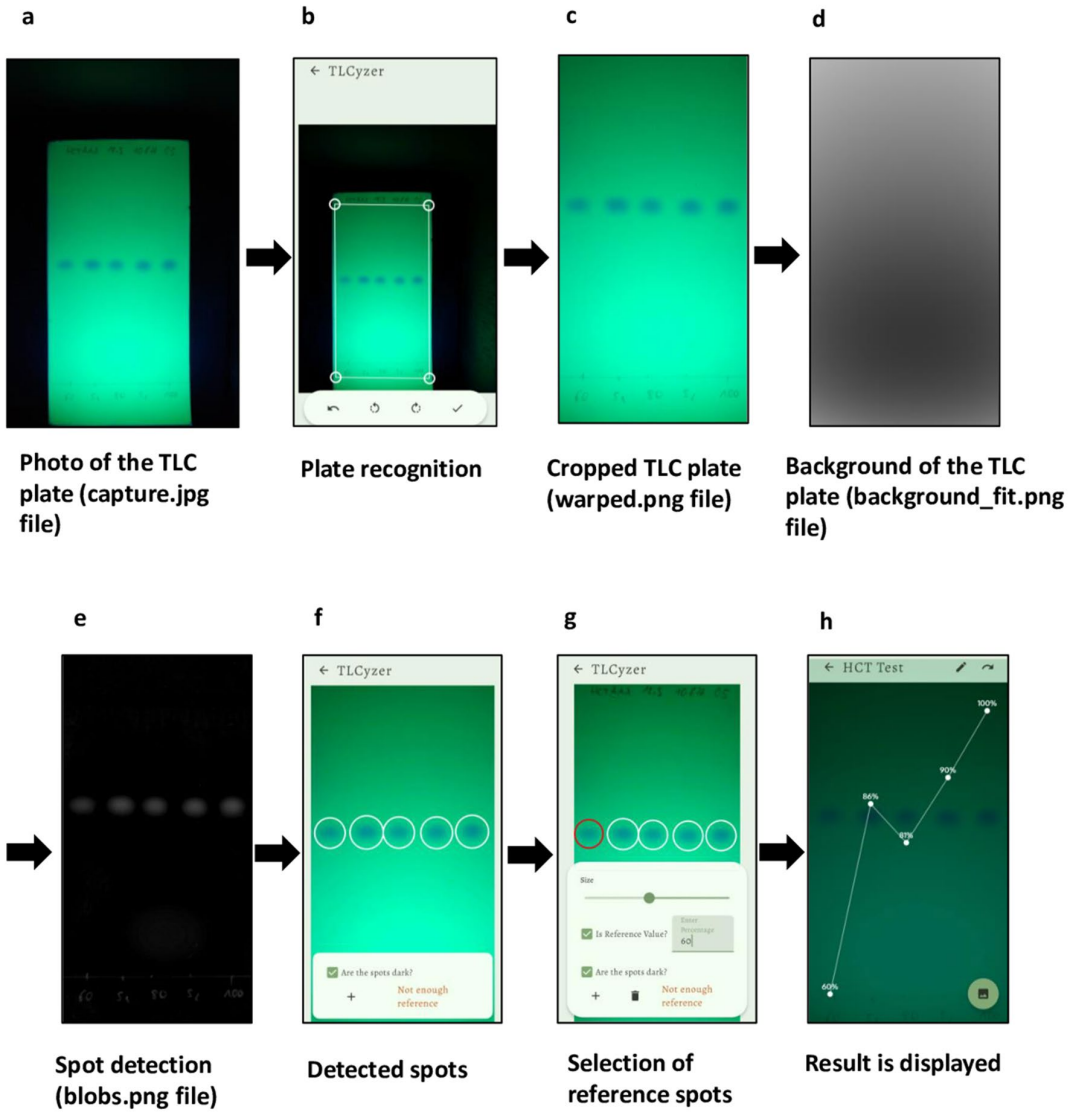
Multi-stage capture and processing pipeline of a photo of a TLC plate in the TLCyzer imaging application. The TLC plate is photographed (**a**) and the outlines of the plate are defined, perspectively warped, and cropped (**b**,**c**). The background is then fitted on the result and removed from the grayscale input (**d**). The result leaves only the blobs, which are detected by thresholding and connected component analysis (**e**). The now detected spots (**f**) are then integrated, and content value (= percentage) is manually entered for each reference spot (**g**). By fitting a linear function, the contents (= percentages) of the unknown samples are evaluated (**h**). A spotting pattern with three reference solutions (60, 80 and 100%) was used in this TLC plate (see Supplementary Fig. S1).

A new image processing algorithm, named “TLCyzer” was developed for this study. Details of the algorithm are described in the Methods section, and the general processing steps are visualized in Fig. 2a–h. Due to the high-performance Rust^47^ implementation the entire processing and analysis of the image can be run on any modern smartphone with short analysis times, aiding the practical on-site capture and analysis setup. The image analysis can be compiled for various operating systems and future ports of the application to Apple’s iOS or Windows systems are easily possible without any modification to the analysis. The analysis will remain consistent between various operating systems and devices.

The TLCyzer app is available free of charge as GPL open-source software (see “Methods”). Instructions for download and use of the app are provided in Supplementary Fig. S3. For the evaluation of a TLC photo with the app, manual user inputs are required for: (i) entering the name or number of the sample; (ii) cropping of the photo, i.e. correct positioning of the four corner points of the image to be evaluated; (iii) if necessary, correction of the automatic detection of the TLC spots; (iv) if necessary, deletion of any unwanted contaminant spots which may have been automatically detected by the app; (v) defining which of the spots are references, and entering their respective concentrations. The app allows the use of two or more reference solutions of any concentration, enabling the user to tailor the analytical procedure to her/his needs.

Tapping the appropriate symbol on the smartphone screen starts the calculation, which takes less than 1 s. The results for all sample and reference spots are subsequently displayed on the screen (Fig. 2h and Supplementary Fig. S3). Intentionally, results are given on the smartphone screen only as integer percentage values in order to avoid exaggerated expectations about the precision of this low-cost screening tool.

Folders containing the original photos and all data generated in the evaluation are saved on the smartphone. The evaluation does not require an internet connection, which is important in low-resource settings where access to the internet is often limited or unreliable. However, once an internet connection is available, the folders can be shared as ZIP files (size approximately 5 MB) using instant messaging apps (like WhatsApp or Signal) or e-mail, and uploaded to a cloud (Supplementary Fig. S3). This enables rapid sharing of TLC photos and of analysis results between personnel in the field and senior staff, as well as re-evaluation of TLC photos of suspicious samples by scientifically trained researchers in any geographical location. Of course, the ZIP files can be uploaded from the smartphone to a computer using a cable or Bluetooth connection. These files contain the calculated percentage values with several digits after the decimal point.

### Requirements and costs of medicine analysis with the TLCyzer app

Requirements and costs for medicine analysis with the GPHF Minilab have been evaluated previously^31^. Quantitative medicine analysis with the TLCyzer app requires, in addition to a GPHF Minilab, the above-described box for photo-taking. This has been produced by a German workshop (see “Methods”) for 69 € (78 US$) per box. A local carpenter in Mutare, Zimbabwe, produced a single box even for 36 US$. This compares favourably to the 130 US$ stated by Yu et al.^41^ as cost of their 3D-printed box. Furthermore, a smartphone with rear-facing camera is required, which must be Android-based for the current version of the app. In the present evaluation, TLC photography and image analysis were carried out with a low-priced smartphone model (see “Methods”), purchased for 250 € (284 US$). Use of the app does not require any further equipment or consumables. Provided that the smartphone is charged, and batteries for the UV lamp of the GPHF Minilab are available, no power connection is required.

### Accuracy and repeatability of quantification with the TLCyzer app

The accuracy (“trueness”) of an analytical procedure is the closeness of the test result to the true value, and should be reported as percent recovery of known amounts of the analyte in the sample^46^. Precision is the degree of agreement of individual test results when the procedure is applied repeatedly to multiple samplings of a homogeneous sample, and is usually expressed as standard deviation (SD) or relative standard deviation (RSD). “Repeatability” refers to the precision of repetitions carried out within a short period of time, by the same person and using the same equipment. In contrast, “intermediate precision” ﻿﻿expresses within-laboratory variations, e.g. between analyses carried out on different days, using different equipment, or by different persons. Both repeatability and intermediate precision were assessed in the present performance evaluation.

**Figure 3:**
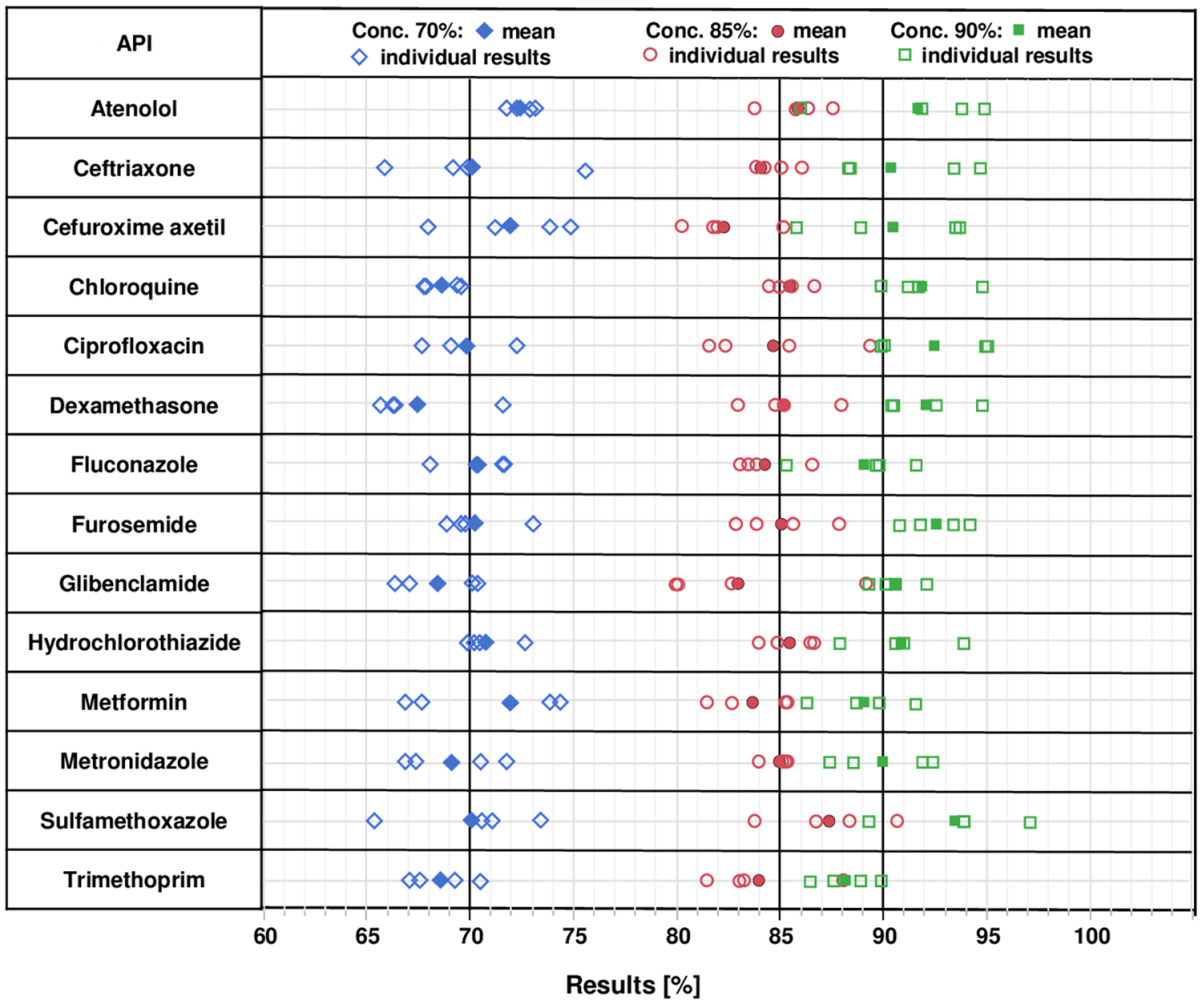
Individual and mean (n = 4) results of the quantitative determination of 14 active pharmaceutical ingredients with the TLCyzer app. For each API, three test solutions containing 70%, 85% and 90% of the standard concentration given in Table 1 were prepared and analysed four times by TLC and image analysis (see “Methods”). These results are further evaluated in Table 2, and the numerical values of all individual measurements are listed in Supplementary Table S2.

14 APIs (Table 1) representing both medicines against infectious diseases and against non-communicable diseases were selected based on (i) their importance for health care in Africa^36,48^, (ii) the availability of monographs for their analysis in the GPHF Minilab manual^49^, and (iii) the possibility of their detection under UV light. As explained in the “Intermediate precision” section, from each API three solutions of different concentrations were prepared and spotted onto two lanes of each of two TLC plates, next to appropriate reference solutions. The APIs sulfamethoxazole and trimethoprim were combined in the solutions, in accordance with their fixed combination in cotrimoxazole preparations. Representative photos of the TLC analysis of all 14 APIs are depicted in Supplementary Fig. S4. The sample spots were quantified using the TLCyzer app. The resulting values are displayed graphically in Fig. 3. All individual measurements are listed in Supplementary Table S2, and the results are summarized in Table 2.

**Table 1:** Investigated active pharmaceutical ingredients and concentration of their “100% reference standard solutions” according to the GPHF Minilab manual^49^. Following appropriate sample preparation procedures given in the GPHF Minilab manual for finished pharmaceutical products of different strengths, this “100%” concentration is equivalent to the concentration of a solution obtained from a sample containing 100% of the declared amount of the API.

API	Concentration of “100% reference standard solution”	Solvent
Atenolol	5 mg/ml	Methanol
Ceftriaxone sodium in powder for injections (= ceftriaxone disodium salt hemiheptahydrate)	0.5 mg/ml^a^	Water:methanol (1:10)
Cefuroxime axetil	1.25 mg/ml^b^	Methanol
Chloroquine phosphate	1.5 mg/ml^c^	Water
Ciprofloxacin HCl	0.625 mg/ml^c^	Aqueous acetic acid (9.6%):methanol (1:8)
Dexamethasone	1 mg/mL	Methanol
Fluconazole	10 mg/ml	Methanol
Furosemide	1.25 mg/ml	Acetone
Glibenclamide	2 mg/ml	Acetic acid:methanol (1:20)
Hydrochlorothiazide	2 mg/ml	Acetone
Metformin HCl	4 mg/ml	Methanol
Metronidazole	5 mg/ml	Methanol
Cotrimoxazole (sulfamethoxazole and trimethoprim)	5 mg/ml and 1 mg/ml	Methanol

^a^Calculated as free acid; ^b^calculated as free cefuroxime; ^c^calculated as free base.

**Table 2:** Repeatability and accuracy of the quantitative determination of 14 active pharmaceutical ingredients with the TLCyzer app. The results of all 168 individual measurements are displayed graphically in Fig. 3, and their numerical values are listed in Supplementary Table S2.

API	Conc. (%)	Mean (%) (n = 4)	SD (%) (n = 4)	Relative SD [%] (n = 4)	Relative SD [%] (n = 12)	Recovery mean (n = 4) [%]
Atenolol	70	72.5	0.59	0.81	2.33	103.5
	85	85.9	1.59	1.85		101.1
	90	91.7	3.97	4.33		101.8
Ceftriaxone	70	70.1	3.99	5.68	3.50	100.2
	85	84.1	1.00	1.18		99.8
	90	90.4	3.31	3.63		101.3
Cefuroxime axetil	70	72.0	3.09	4.29	3.70	102.9
	85	82.3	2.13	2.59		96.8
	90	90.5	3.82	4.23		100.5
Chloroquine	70	68.7	0.93	1.36	1.59	98.1
	85	85.5	0.95	1.11		100.5
	90	91.9	2.11	2.30		102.1
Ciprofloxacin	70	69.8	1.93	2.77	3.34	99.7
	85	84.7	3.53	4.17		99.6
	90	92.5	2.86	3.09		102.8
Dexamethasone	70	67.5	2.75	4.08	2.92	96.4
	85	85.2	2.07	2.43		100.3
	90	92.1	2.08	2.26		102.3
Fluconazole	70	70.4	1.68	2.39	2.40	100.6
	85	84.3	1.54	1.82		99.1
	90	89.1	2.68	3.01		99.0
Furosemide	70	70.3	1.82	2.59	2.28	100.5
	85	85.1	2.20	2.58		100.1
	90	92.6	1.54	1.66		102.8
Glibenclamide	70	68.5	2.04	2.98	3.17	97.9
	85	83.0	4.34	5.23		97.6
	90	90.6	1.17	1.29		100.6
Hydrochloro-thiazide	70	70.8	1.27	1.80	1.98	101.2
	85	85.5	1.24	1.45		100.6
	90	90.9	2.46	2.70		101.0
Metformin	70	72.0	3.45	4.80	3.21	102.8
	85	83.7	1.94	2.32		98.5
	90	89.1	2.25	2.53		99.0
Metronidazole	70	69.2	2.38	3.44	2.33	98.8
	85	85.0	0.66	0.77		100.0
	90	90.0	2.50	2.77		100.0
Sulfamethoxazole	70	70.1	3.38	4.82	3.84	100.2
	85	87.4	2.91	3.33		102.8
	90	93.5	3.17	3.39		103.9
Trimethoprim	70	68.6	1.53	2.22	2.48	98.0
	85	84.0	2.86	3.41		98.8
	90	88.2	1.61	1.82		97.9
Mean				2.79		100.3

Accuracy, expressed as mean recovery for each of the test solutions, was 100.3% on average (range 96.8–103.9%). Repeatability, expressed as RSD, was 2.79% on average, and ranged from 1.59 to 3.84% for the 14 tested APIs (Table 2). The highest RSD was observed for sulfamethoxazole. Sulfamethoxazole gives quite large spots, since the concentrations given in the GPHF Minilab manual are optimized for parallel detection of both sulfamethoxazole and the minor component trimethoprim in the analysis of cotrimoxazole preparations. The large sulfamethoxazole spots were often not recognized automatically by the TLCyzer app and required manual spot detection.

Contrary to our expectations, APIs giving only faint spots in the TLC analysis, such as atenolol and trimethoprim (Supplementary Fig. S4), did not show higher RSDs (Table 2).

### Intermediate precision

The following intra-laboratory variations were introduced: photographs of the TLC plates were taken on two different days; a total of three different photos were taken of every plate; the TLCyzer evaluation of these photos was carried out by three different investigators, each one using a different smartphone model (see “Methods”). As recommended by the ICH guidelines^46^, the effects were evaluated within an experimental matrix design using a random combination of the above-mentioned variations, as explained in the “Methods” section. Table 3 shows a summary of the results, and Supplementary Table S3 lists the results of each individual measurement. The most experienced investigator (C.H.), who had already carried out the investigation of the repeatability, obtained results with an RSD of 3.50%, slightly higher than the RSD of 2.79% observed in the repeatability experiment and likely to reflect the expected effect of introduced variations. The results of the two other investigators (undergraduate students Y.W. and J.G.) showed higher RSDs (4.32% and 5.45%, respectively) than those of investigator C.H., most likely reflecting the lesser degree of their training in the use of the TLCyzer app. As shown in Table 3, the average RSD including all three investigators with all 14 APIs resulted as 4.46%.

**Table 3:** Intermediate precision of the quantitative determination with the TLCyzer app. The results of all 336 individual measurements are listed in Supplementary Table S3.

API	Mean (%) (n = 24; true value 90%)	SD (%)	RSD (%)
Atenolol	88.5	4.38	4.94
Ceftriaxone	88.1	2.82	3.20
Cefuroxime axetil	91.2	4.57	5.01
Chloroquine	91.8	3.67	4.00
Ciprofloxacin	90.5	4.55	5.03
Dexamethasone	90.8	4.04	4.45
Fluconazole	89.6	3.22	3.60
Furosemide	91.1	3.58	3.93
Glibenclamide	90.5	2.87	3.17
Hydrochlorothiazide	90.4	4.07	4.50
Metformin	89.1	3.70	4.15
Metronidazole	91.6	4.32	4.71
Sulfamethoxazole	88.3	7.08	8.02
Trimethoprim	91.9	3.38	3.68
Mean	90.2	4.02	4.46

Among the 14 APIs, the highest RSD (8.02%) was again observed for sulfamethoxazole. Among all the 336 individual measurements, the recovery results ranged from 84.9 to 113.7% of the true value, i.e. over a wider range than the 168 individual measurements for repeatability (93.4–107.0%), consistent with the expected effect of the introduced variations.

### Linearity

**Figure 4:**
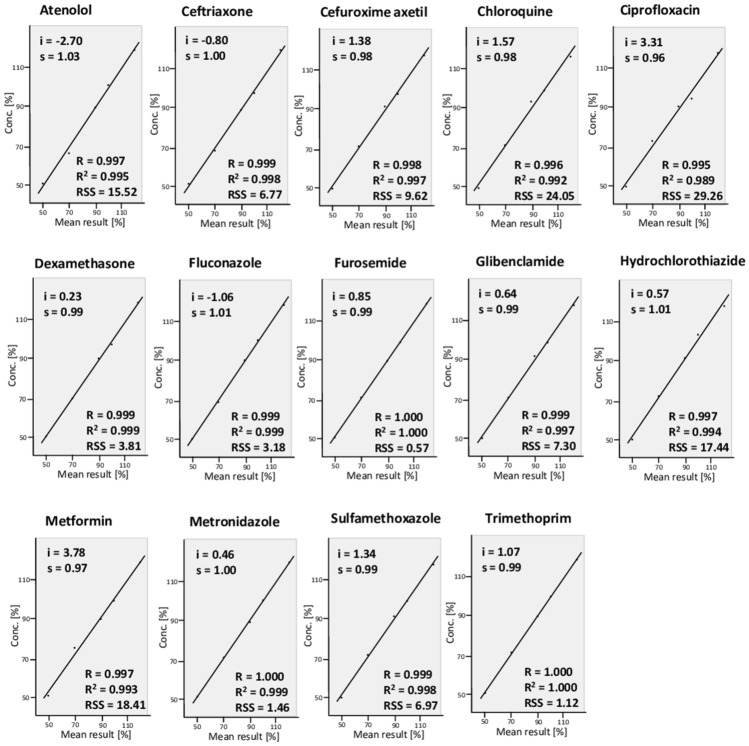
Linearity plots of the quantitative determination with the TLCyzer app. Solutions containing 50–120% of the standard concentrations given in Table 1 were prepared, and each was analysed in triplicate. The y-intercepts (i), slopes of the regression lines (s), the correlation coefficients (R), determination coefficients (R^2^) and residual sums of squares (RSS) are indicated. The individual measurements are listed in Supplementary Table S4.

Linearity is the ability to obtain test results that are proportional to the concentration of the analyte in the sample across a given range^46^. For each of the 14 APIs, solutions of five different concentrations were prepared and analysed as described in the “Methods” section. As recommended by the ICH guideline^46^, plots of the results are depicted in Fig. 4 with y-intercepts (i), slopes of the regression lines (s), the correlation coefficients (R), determination coefficients (R^2^) and residual sums of squares (RSS). All individual test results are listed in Supplementary Table S4. The test results were proportional to the concentrations of the analyte for all 14 APIs in the investigated range, with determination coefficients R^2^ between 0.989 and 1.00. The data in Supplementary Table S4 again confirm the accuracy of the method (mean recovery 100.1%), and its precision (average RSD 1.99%). Among all the 210 individual measurements listed in Supplementary Table S4, the recovery results ranged from 92.1 to 109.0% of the true value.

### Range

The above data on linearity prove that the quantification with the TLCyzer app has suitable levels of accuracy, precision and linearity for all 14 investigated APIs in the range from 50 to 120% of the declared API content of a pharmaceutical product, i.e. in the range most relevant for the assessment of medicine quality. To investigate whether also lower amounts of API can be accurately quantified, three test solutions of sulfamethoxazole and trimethoprim containing only 12–15% of the standard concentrations were prepared and analyzed as described in the “Methods” section. A photo of one of the resulting TLC plates is included in Supplementary Fig. S4. The spots of trimethoprim are very faint under these conditions. However, TLCyzer analysis of these photos still quantified trimethoprim correctly with a mean recovery of 100.9%. The observed RSD of 5.2% (n = 12) was approximately twice as large as the RSD of 2.5% (n = 12) obtained with the higher trimethoprim concentrations (Table 2).

### Robustness

The robustness of an analytical procedure is a measure of its capacity to remain unaffected by small deliberate variations in procedural parameters, and provides an indication of the procedure’s suitability during normal use^21,46^. Four different APIs (chloroquine, dexamethasone, hydrochlorothiazide and metformin) were selected to for the evaluation of robustness. As explained in the “Methods” section, they were analysed under the standard conditions, but also under seven modified conditions representing small deliberate variations (Table 4): including or excluding different parts of the TLC photos (Supplementary Fig. S1); using manual spot detection, rather than automatic spot detection by the app; shifting the UV lamp out of its central position in the box (2 cm to the left); using batteries with low charge (ca. 1.3 V) for the UV lamp; using a different model of the box for photo-taking (i.e. the box depicted in Supplementary Fig. S5a); and using a different smartphone for TLC photography and TLCyzer evaluation (see “Methods”). The results are summarized in Table 4, and the individual results of all measurements are listed in Supplementary Supplementary Table S5. Overall, accuracy and precision were hardly affected by the deliberate modifications: the values for mean recovery (100.9%) and for average RSD (2.60%) were nearly identical to those determined for these four APIs under standard conditions (recovery 100.2%; RSD 2.43%; calculated from the values shown in Table 2). Only one modification led to a conspicuously increased RSD (3.99%), namely the shift of the UV lamp out of its central position which results in a stronger illumination of the left side of the TLC plate. Indeed, closer examination of the results showed that under these conditions average recovery for the left sample spot was higher by 5.8% than for the right sample spot on the same plate. This shows the importance of the central positioning of the UV lamp.

**Table 4:** Robustness of the quantitative determination with the TLCyzer app. The numerical values of all 192 individual measurements are listed in Supplementary Table S5.

Modification	API	Mean (n = 6; true value 90%)	Recovery of the respective API (n = 6) (%)	RSD of respective API (n = 6) (%)	Mean recovery of the four APIs (n = 24) (%)	Mean RSD of the four APIs (n = 24) (%)
Standard conditions	Chloroquine	92.1	102.4	2.67	101.0	2.17
	Dexamethasone	89.1	98.9	2.12		
	Hydrochlorothiazide	93.1	103.4	1.12		
	Metformin	89.3	99.2	2.75		
Cropping: full plate with labelling (see Supplementary Fig. S1b)	Chloroquine	93.7	104.1	1.81	102.7	2.17
	Dexamethasone	91.9	102.1	3.31		
	Hydrochlorothiazide	93.3	103.6	2.12		
	Metformin	90.7	100.8	1.44		
Cropping: without labelling (see Supplementary Fig. S1b)	Chloroquine	91.5	101.7	3.63	100.3	2.52
	Dexamethasone	88.9	98.8	2.42		
	Hydrochlorothiazide	91.8	102.0	1.82		
	Metformin	89.0	98.9	2.22		
Manual spot detection	Chloroquine	93.7	104.1	1.84	102.2	2.65
	Dexamethasone	91.9	102.1	3.51		
	Hydrochlorothiazide	92.6	102.9	3.57		
	Metformin	89.8	99.8	1.66		
UV lamp not central	Chloroquine	92.4	102.6	4.51	102.2	3.99
	Dexamethasone	93.2	103.6	4.39		
	Hydrochlorothiazide	92.9	103.2	5.07		
	Metformin	89.4	99.3	2.00		
Low battery charge of UV lamp	Chloroquine	89.3	99.2	1.08	99.0	3.04
	Dexamethasone	87.7	97.4	2.36		
	Hydrochlorothiazide	88.5	98.3	5.11		
	Metformin	90.9	101.0	3.63		
Different box for photo-taking (see Supplementary Fig. S5a)	Chloroquine	90.4	100.4	1.97	100.3	1.37
	Dexamethasone	89.7	99.6	1.29		
	Hydrochlorothiazide	91.2	101.3	1.06		
	Metformin	89.8	99.7	1.16		
Different smartphone model for photography and TLCyzer analysis (see “Methods”)	Chloroquine	88.2	97.9	2.90	99.3	2.88
	Dexamethasone	89.5	99.4	2.86		
	Hydrochlorothiazide	89.4	99.3	2.82		
	Metformin	90.4	100.4	2.95		

Among all 192 individual measurements in this experiment, the recovery values ranged from 91.1 to 110.2%, with the highest deviation observed for a measurement carried out with a non-central position of the UV lamp.

### Specificity: analysis of finished pharmaceutical products

Specificity is the ability to assess the analyte, with a suitable level of accuracy and precision, also in the presence of other components that may be present^21,46^. The specificity of the GPHF Minilab in the qualitative identification of APIs has been investigated previously^13,31,34,36^. In the present evaluation, tablets of ciprofloxacin, dexamethasone, hydrochlorothiazide and metronidazole were obtained from the pharmacy of the Tübingen University Hospital, and were analysed to evaluate accuracy and precision of the quantification with the TLCyzer app in the presence of matrix components (see “Methods”). In addition, the API contents of the four products were determined by HPLC according to the methods of USP 42. The contents were found to be compliant with USP 42 specifications (Supplementary Table S6).

As shown in Supplementary Table S6, accuracy of the quantification with the TLCyzer (mean recovery 99.5%) was only slightly lower, and the average RSD (3.38%) was only slightly higher, than the values determined with the respective solutions of the pure APIs (i.e. mean recovery 100.2%; average RSD 2.64%; calculated from the values in Table 2). This suggests that the quantitative evaluation with the TLCyzer app was not affected by matrix components of the investigated tablets.

### Use of the TLCyzer app in the analysis of substandard medicines collected in the DR Congo

As a first experiment to evaluate the performance of the app in the analysis of actual substandard medicines, we tested two samples of substandard tablets, i.e. ciprofloxacin 500 mg tablets and metronidazole 250 mg tablets, which we had collected in the course of a medicine quality study in the DR Congo^36^. These were analysed by HPLC according to the methods of USP 42, resulting in a content of only 83.4% and 86.7% of the declared amount of the API, respectively^36^. Therefore they failed the specifications of USP 42 which demands a content of 90–110% of the declared amount. These two samples were now analysed with the TLCyzer app. The results (84.2% for the ciprofloxacin sample; 86.0% for the metronidazole sample) were in remarkably close agreement with the values determined by HPLC). For the substandard ciprofloxacin (API content 83.4% of the declared amount), all 12 individual measurements obtained with the TLCyzer app showed a content lower than 90% of the declared amount of the API (Supplementary Table S6), correctly identifying the sample as OOS. For the substandard metronidazole (API content 86.7%), 11 of the 12 individual measurements correctly identified the sample as OOS, while one measurement was above 90% and incorrectly suggested the sample to be within specification. For the four good-quality products obtained from the Tübingen University hospital pharmacy, 46 of the 48 individual measurements correctly identified the samples as in-specification. However, for hydrochlorothiazide tablets (API content 93.3%), two individual measurements were below 90%, incorrectly suggesting the sample to be OOS (Supplementary Table S6). A correct identification of all substandard products as OOS, and all good-quality products as in-specification, was obtained when the mean results from two different TLC plates, or just two different photos of the same TLC plate, were used for calculation.

### Simplified spotting pattern: 80% and 100% reference solutions only

Despite the use of simple, low-cost equipment (standard TLC plates; manual spotting of sample and reference solutions; low-cost smartphone camera), the quantification with the TLCyzer app had shown quite good accuracy and precision, exceeding our original expectations. This encouraged us to test a further simplification, i.e. the use of only two reference solutions, containing 80% and 100% of the standard concentrations shown in Table 1, respectively. This allows one to strictly follow the instructions of the GPHF Minilab manual^49^ both in the preparation of the reference solutions and in the spotting pattern on the TLC plate (Supplementary Fig. S1). The solutions prepared for the above described investigation of four good-quality products and two substandard products were used for this experiment. The results from the good-quality medicines (98.9% recovery; 3.60% average RSD; see Supplementary Table S7) were very similar to those obtained with the previous procedure using three reference solutions (99.5% recovery; 3.38% average RSD; see Supplementary Table S6). The same was true for the results of the substandard products. The discrimination between in-specification and OOS medicines was even more precise using the simplified spotting pattern: for the four good-quality products, all 48 individual measurements correctly identified the samples as in-specification. For the two substandard products from the DR Congo, 23 out of the 24 individual measurements correctly identified the sample as substandard; only for the metronidazole tablets (API content 86.7% of the declared amount), one individual measurement was slightly above the 90% threshold (i.e. 90.3%).

## Discussion

The development of simple, low-cost screening tools which allow a rapid quantification of the API content of medicine samples in low-resource settings still represents a challenge (see “Introduction”). The present study provides a proof of principle that such quantitative screening analysis is possible using thin-layer chromatography according to the procedures of the GPHF Minilab^49^ followed by photography of the TLC plates and image analysis using the open-source TLCyzer app developed here. Despite the use of low-cost, conventional TLC plates, manual spotting of samples and references, and low-cost smartphones for photography and image analysis, very good accuracy and reasonable precision was achieved. The determined repeatability of the individual measurements (average RSD 2.79%) suggests that medicine samples containing 85.9% or less of the stated amount of the API can be identified with 95% confidence as OOS, assuming that specifications demand a content of ≥ 90% for this medicine^50^. This is similar to the precision reported by Yu et al.^41^ for the evaluation of a related method. Following the recommendations of international guidelines^21,46^, we also investigated intermediate precision, resulting in an average RSD of the individual measurements of 4.46%. This value suggests that medicine samples containing 83.4% (or less) of the stated amount of the API can be identified with 95% confidence as OOS (assuming specifications demanding ≥ 90%). Figure 3 visually illustrates the power and the limitations of the TLCyzer method in the discrimination between different quantities of APIs. This discriminative power is a clear improvement over the visual evaluation of TLC results in the conventional GPHF Minilab procedure, which has been found to identify as OOS only 43%^36^ (or even only 24%^27^) of samples containing 80% or less of the declared amount of the API. Notably, the two substandard medicines from the DR Congo which in the present study were readily identified as OOS by TLCyzer analysis had both escaped identification as substandard medicines in the conventional GPHF Minilab analysis used in the previous study^36^.

Obviously, the precision of the TLCyzer quantification method can be further improved if instead of individual results of only one measurement the mean values of multiple determinations of the same sample are used for calculation. E.g. the RSD determined in the repeatability experiment (Supplementary Table S2) is reduced from 2.79 to 1.54% if the mean values of the four individual measurements for each sample is used in the calculation. In analytical practice, a compromise between the advantage of increased precision and the disadvantage of increased workload of multiple determinations will have to be searched. In the user instructions in Supplementary Fig. S3, we recommended the use of the mean value of two determinations on a single TLC plate.

Optimal precision of the quantification with the TLCyzer app requires optimal handling in the preceding TLC analysis. This begins with complete extraction of the API from the investigated product; in the present evaluation, this step was not found to be problematic. All volumes for the preparation of sample and reference solutions must be measured precisely, as well as the 2 µl aliquots spotted onto the TLC plates. Reference solutions must be free of degradation products and contaminants. Saturation of the TLC chamber with solvent vapours must be ensured prior to inserting the loaded TLC plate. All these procedures are excellently explained and illustrated in the GPHF Minilab manual^49^, but still require hands-on training in the laboratory, especially for non-scientific staff.

In addition, the evaluation with the TLCyzer app requires complete drying of the TLC plate after development, for at least 30 min at room temperature. The proper standardization of TLC plate photography is largely ensured by the box depicted in Fig. 1. The image analysis with the smartphone requires precise user operations on the smartphone screen (see Supplementary Fig. S3), and these require training especially in the case of non-scientific staff. Such training is provided ideally face-to-face, otherwise online. In addition to the step-by-step instructions given in Supplementary Fig. S3, we plan to prepare instructional videos for the use of the app in the future.

The procedures for GPHF Minilab analysis have been tested and improved over decades, and they now represent mature analytical techniques. In contrast, the TLCyzer app was developed in this study, without extramural funding, and further improvements of this app are certainly possible and desirable. The app is provided as an open-source software^51^, and we highly welcome competent individuals and organizations to contribute to its further development. Improvements should be based especially on needs identified in field tests of the technology in low-resource settings. Once the COVID-19 pandemic allows, we hope to initiate such field tests in cooperation with partner organizations in Africa. Among other questions, future tests may investigate which of the two spotting patterns depicted in Supplementary Fig. S1 proves to be more suitable in practice. Until additional studies, and subsequent improvements of the method, have been implemented, we suggest not to use the TLCyzer app without close scientific supervision.

The methodology presented in this study certainly has potential for medicine quality screening in low-resource settings. GPHF Minilabs are already widely available. The box for TLC plate photography depicted in Fig. 1 can be produced locally (or ordered from a workshop in Germany, see “Methods”), smartphones are universally available also in LMICs, and the TLCyzer app is provided free of charge over the internet^51^. Therefore, this technology can be implemented with hardly any additional investment wherever a Minilab is in place. GPHF Minilab monographs are currently available for 107 different APIs and for their common fixed combinations, and UV detection is possible for the majority of the APIs, making TLCyzer analysis applicable to a wide range of products. TLC analysis is specific for the API, not for the respective brand of the medicine, and therefore it does not require libraries of data to be established and maintained for a multitude of commercial products, as is the case e.g. for NIR or Raman spectroscopic methods in medicine analysis.

The role and the limitations of screening tools in medicine quality assurance in low-resource settings need to be considered responsibly. Compliance with pharmacopeial standards, which is a prerequisite e.g. for medicine licensing in a given country, can only be proven by pharmacopeial methods, and cannot be reliably assured by simple, low-cost screening tools. Unfortunately, however, the lack of laboratory capacity in many LMICs does not allow a sufficient number of compendial analyses, and this lack contributes to the entry of seriously substandard and of falsified medicines into the supply chains, as documented by WHO^1,5^. This severely affects the health of the population. In this situation, screening tools like the GPHF Minilab and the TLCyzer app can provide the possibility to screen large numbers of medicine samples and to forward those which appear suspicious to compendial analysis. This allows the fully equipped laboratories to focus their precious resources onto the most serious problems. Simple low-cost tools also empower personnel working on various levels of the health care system to take an active role in medicine quality assurance, improving not only effective identification and removal of dangerous products, but also increasing the awareness of the issue of medicine quality. Screening tools are therefore fundamentally important to achieve universal health coverage and access to safe, effective, quality and affordable essential medicines, as proposed in target 3.8 of the Sustainable Development Goals^52^.

### Limitations of this study

This study is a laboratory performance evaluation of the quantitative analysis using the TLCyzer app, with most of the analytical work carried out by an experienced researcher; field studies with non-scientific personnel in LMICs still have to be carried out. The evaluation of specificity was limited to analysis in the presence of matrix components of tablets; specificity of the quantification of APIs in the presence of common contaminants and degradation products was not investigated, and such compounds may not be separated from the APIs in some TLC analysis methods. The TLCyzer software, and the instructions for its use (Supplementary Fig. S3) are so far only available in English; translations into other languages are desirable for use in non-anglophone countries.

## Methods

### Investigated active pharmaceutical ingredients and finished pharmaceutical preparations

Secondary reference standards of 14 APIs (Table 1) were obtained from Sigma Aldrich (St. Louis, MO, USA) and from EDQM (European Directorate for the Quality of Medicines & Health Care, Strasbourg, France). Following the recommendations given in the GPHF Minilab manual^49^, reference solutions of these APIs were prepared using the solvents listed in Table 1.

Tablets of dexamethasone, hydrochlorothiazide, ciprofloxacin and metronidazole were obtained from the Tübingen University Hospital Pharmacy. Brands and manufacturers are shown in Supplementary Table S6. The batch numbers were J71155, 16L008, 45333 and 72201, respectively. Two substandard samples of ciprofloxacin and metronidazole tablets had been identified in a medicine quality study of our group in the DR Congo^36^. Brands and manufacturers stated on their label are shown in Supplementary Table S6.

### Thin-layer chromatography

TLC plates of 5 × 10 cm size with fluorescence indicator (TLC Silica gel 60 F_254_; Merck KGaA, Darmstadt, Germany, or ALUGRAM^®^ Xtra SIL G UV254, Macherey–Nagel GmbH & Co. KG, Düren, Germany) were used. Sample and reference solutions were applied to these plates manually, using 2 µl capillaries (minicaps^®^, Hirschmann Laborgeräte GmbH & Co. KG, Eberstadt, Germany). Two different spotting patterns were evaluated, shown in Supplementary Fig. S1. Plates were developed as described in the respective monographs of the GPHF Minilab Manual^49^. The composition of the mobile phases is given in Supplementary Table S1. After development, the plates were left to dry completely for at least 30 min at room temperature. The spots of the APIs were detected under UV light using a battery-operated hand lamp (MINI-UV Test Lamp, 256 nm, Prinz Verlag GmbH, Passau, Germany). Storage of the developed plates for up to three days, protected from light and humidity, did not affect the quantitative evaluation for the investigated APIs. No chemical staining of the spots was carried out in this study.

### Photography of TLC plates

Photos of the TLC plate were taken using the box depicted in Fig. 1 and Supplementary Fig. S2, produced by a workshop of the University of Tübingen. In the evaluation of robustness, also a box produced in a different workshop (Pidinger Werkstätten, Piding, Germany) was used.

A Motorola Moto G7 smartphone (12 MP camera; Motorola Solutions, Chicago, IL, USA) was used for photography and image analysis. In the evaluation of robustness, also a Fairphone 3 (12 MP camera; Fairphone B.V., Amsterdam, The Netherlands) was used for comparison. The original photos were stored as JPEG files on the smartphones.

### TLC imaging application

The developed application follows a split architecture, where the user interface (UI) is developed in Kotlin^53^ for the Android operating system and the image analysis is performed in a high-performance Rust^47^ implementation. The app has a capturing mode implemented which automatically picks a focus point and performs an auto-exposure operation of the smartphone. After a JPEG photo of approximately 12 megapixel is taken, the first step is to detect the TLC plate in the image (Fig. 2). This plate detection is performed in an image downscaled by a factor of four, using thresholding and line detection in Hough space^54^. The user can set the position of the four corner points of the image in the application, excluding areas not belonging to the TLC plate. The selected corner points are then used to remove the perspective warp and crop the image to contain the plate.

For an accurate spot integration, only the intensity of the spot needs to be considered. Compared to Yu et al.^41^ our method does not require multiple input images of a blank TLC plate for the background illumination subtraction. For the fitting and spot integration, the image is converted to grayscale: $Y = 0.2126 \times R + 0.7152 \times G + 0.0722 \times B$, where R, G, B respond to linear red, green and blue channel, respectively. We remove the lighting by fitting the 15 coefficients ${a}_{j}$ of the two-dimensional quartic polynomial function:

$$f\left(x, y\right)={a}_{1}{y}^{4}+{a}_{2}x{y}^{3}+{a}_{3}{{x}^{2}y}^{2}+{a}_{4}{x}^{3}y+{a}_{5}{x}^{4}+{a}_{6}{y}^{3}+{a}_{7}x{y}^{2}+{a}_{8}{x}^{2}y+{a}_{9}{x}^{3}+{a}_{10}{y}^{2}+{a}_{11}xy+{a}_{12}{x}^{2}+{a}_{13}x+{a}_{14}y+{a}_{15},$$

where x and y define the image pixel coordinate. Here, we leverage the linregress library of Kacprowski et al.^55^. The result is a smooth approximation of the illumination. As the background fitting on the full resolution images is time-consuming on a smartphone processor and the illumination is smooth, we downsample the image by a factor of 4 for this process. The fitted polynomial illumination model is then subtracted from the image, leaving just the spots and pencil markings.

The resulting image is then automatically thresholded based on the remaining image mean value ${\mu }_{I}$: $$t(v)=\left\{\begin{array}{c}1\, if\, v \ge {\mu }_{I}\\ 0\, otherwise\end{array}\right.$$, and a connected component algorithm labels all remaining areas. Based on the shape and size, all components are filtered, and only the spots remain. Here, shapes with a wide or tall aspect ratio, small shapes, and shapes larger than a quarter of the image are filtered. The center of the spots is calculated by calculating the mean of the pixel coordinates weighted by the intensity values: $\frac{\sum_{1}^{N}cv}{\sum_{1}^{N}v}$, where c describes the two-dimensional coordinate and v the pixel intensity. The radius is then selected to cover the whole spot. The integral of the intensity values is then calculated by summing the pixel values in this circular region. We found that the robustness is improved by only selecting the top 15% of the pixels in the spot region, therefore this procedure was followed.

With the user-provided reference API concentration percentages and the corresponding integrant values, a linear model is fit using linear regression, that maps the integrant values to the percentages. The linear model is then evaluated with the sample spots integration values to calculate the API concentration per sample spot.

Results are saved as a ZIP file on the smartphone. For each evaluated photo, a folder is created which contains the raw photo (capture.jpg), the cropped photo (warped.png), the background (background_fit.png), the detected spots (blobs.png) and a text file (capture.json) that indicates the user-defined name of the sample (“agentName”), as well as for every detected spot its position in the image (“x” and “y” values), its “radius”, its “integrationValue” and its final quantitation result given as “percentage”. Pictures of the image files are shown in Fig. 2a,c,d,e.

The application was named TLCyzer (current version 0.3) and can be downloaded as GPL open-source software^51^ from the Google Play Store (Mountain View, CA, USA). Three exemplary TLC photos are provided as .jpg files in the Supplementary Information.

### Performance evaluation

Sample and reference solutions of the 14 APIs were prepared as described above, in the concentrations given in Table 1 and in the “Results” sections. The performance evaluation followed the “ICH guideline on validation of analytical procedures”^46^ and the USP chapter “ < 1850 > Evaluation of screening technologies for assessing medicine quality”^21^. The ICH guideline suggests that accuracy and repeatability should be assessed using a minimum of nine determinations over a minimum of three concentration levels^46^. Therefore, from each API three sample solutions were prepared, containing 70%, 85% and 90% of the standard concentrations shown in Table 1. Each solution was spotted onto two lanes of each of two TLC plates. Reference solution containing 60%, 80% and 100% of the standard concentrations were applied to the other three lanes (see Supplementary Fig. S1). This resulted in 12 determinations for each API. TLC analysis, photography and quantitative evaluation was carried out as described above. The resulting files were downloaded from the smartphone to a computer, and percentage results with one digit after the decimal point were used in the calculations.

To assess intermediate precision, a total of three different photographs of each TLC plate were taken on two different days, and the TLCyzer evaluation of these photos was carried out by three different investigators, each one using a different smartphone model (Motorola Moto G7, Motorola Solutions, Chicago, IL, USA; OnePlus 6 T, OnePlus, Shenzhen, Guangdong, China; and Samsung Galaxy S7, Samsung Electronics Co., Ltd., Suwon, South Korea). Three different photos and three investigators allowed nine possible combinations. On each of the two days, six of these nine combinations were chosen randomly, resulting in 12 combinations for every API, as listed in Supplementary Table S3.

To assess linearity, for each of the 14 APIs solutions containing 50%, 70%, 90%, 100% and 120% of the standard concentrations given in Table 1 were prepared and applied once onto each of three different TLC plates. No mathematical transformation of the results was necessary prior to the regression analysis.

To investigate the method also in a lower concentration range, the test solutions of sulfamethoxazole and trimethoprim used in the repeatability determination were diluted by a factor of six, i.e. now containing only 12.0%, 14.2% und 15.0% of the standard concentrations given in Table 1. They were analyzed in comparison to reference solutions containing 10.0%, 13.3% und 16.7% of the standard concentrations.

To assess robustness, solutions of chloroquine, dexamethasone, hydrochlorothiazide and metformin containing 90% of the standard concentrations (Table 1) were prepared and applied to two spots of each of three TLC plates. Analysis was carried out with small deliberate variations of the conditions, as explained in the “Results” section.

For the investigation of finished pharmaceutical products, tablets were crushed and extracted as described in the GPHF Minilab manual. The resulting solutions were spotted twice onto three different TLC plates. Two photos were taken of each plate and evaluated.

### HPLC analysis

HPLC analysis of finished pharmaceutical products was carried out according to the respective monographs of the USP 2019 (USP 42), using an Agilent 1100 HPLC and 1260 Infinity II HPLC system (Agilent Technologies, Santa Clara, CA, USA). Certified pharmaceutical secondary reference standards were purchased from Sigma-Aldrich (St. Louis, MO, USA).

### Statistical calculations

Statistical evaluations (mean, standard deviation, relative standard deviation, confidence intervals) were performed using JMP 15.0 (SAS GmbH, Heidelberg, Germany).

## Supplementary Information

Supplementary Information.

Supplementary TLC Photo Dexamethasone.

Supplementary TLC Photo Hydrochlorothiazide.

Supplementary TLC Photo Metformin.

## Data Availability

All data generated or analysed during this study are included in this published article and its Supplementary Information files.

## References

[1] WHO. A study on the public health and socioeconomic impact of substandard and falsified medical products. (2017). https://apps.who.int/iris/bitstream/handle/10665/331690/9789241513432-eng.pdf?sequence=1&isAllowed=y (Accessed 29 July 2022).

[2] UNODC. Combating falsified medical product-related crime: a guide to good legislative practices. (2019). https://www.unodc.org/documents/treaties/publications/19-00741_Guide_Falsified_Medical_Products_ebook.pdf (Accessed 29 July 2022).

[3] Petersen A, Held N, Heide L, Difäm - EPN Minilab Survey Group (2017). Surveillance for falsified and substandard medicines in Africa and Asia by local organizations using the low-cost GPHF Minilab. PLoS One.

[4] Lalani M, Kitutu FE, Clarke SE, Kaur H (2017). Anti-malarial medicine quality field studies and surveys: A systematic review of screening technologies used and reporting of findings. Malar. J..

[5] WHO. WHO Global surveillance and monitoring system for substandard and falsified medical products. (2017). https://apps.who.int/iris/handle/10665/326708 (Accessed 29 July 2022).

[6] Risha PG (2008). The use of Minilabs to improve the testing capacity of regulatory authorities in resource limited settings: Tanzanian experience. Health Policy.

[7] Vickers S (2018). Field detection devices for screening the quality of medicines: A systematic review. BMJ Glob. Health.

[8] Chen HH (2021). Cost savings of paper analytical devices (PADs) to detect substandard and falsified antibiotics: Kenya case study. Med. Access Point Care..

[9] Roth L, Biggs KB, Bempong DK (2019). Substandard and falsified medicine screening technologies. AAPS Open.

[10] Pan H, Ba-Thein W (2018). Diagnostic accuracy of Global Pharma Health Fund Minilab in assessing pharmacopoeial quality of antimicrobials. Am. J. Trop. Med. Hyg..

[11] Batson JS (2016). Assessment of the effectiveness of the CD3+ tool to detect counterfeit and substandard anti-malarials. Malar. J..

[12] Opuni KF (2019). Usefulness of combined screening methods for rapid detection of falsified and/or substandard medicines in the absence of a confirmatory method. Malar. J..

[13] Khuluza F, Kigera S, Jähnke RW, Heide L (2016). Use of thin-layer chromatography to detect counterfeit sulfadoxine/pyrimethamine tablets with the wrong active ingredient in Malawi. Malar. J..

[14] Weaver AA, Lieberman M (2015). Paper test cards for presumptive testing of very low quality antimalarial medications. Am. Soc. Trop. Med. Hyg..

[15] Myers NM, Kernisan EN, Lieberman M (2015). Lab on paper: Iodometric titration on a printed card. Anal. Chem..

[16] Kaale E, Risha P, Layloff T (2011). TLC for pharmaceutical analysis in resource limited countries. J. Chromatogr. A.

[17] Kovacs S (2014). Technologies for detecting falsified and substandard drugs in low and middle-income countries. PLoS One.

[18] IDDO. An evaluation of portable screening devices to assess medicines quality for national Medicines Regulatory Authorities. (2018). https://www.iddo.org/external-publication/evaluation-portable-screening-devices-assess-medicines-quality-national (Accessed 29 July 2022).

[19] Bakker-t Hart IME, Ohana D, Venhuis BJ (2021). Current challenges in the detection and analysis of falsified medicines. J. Pharm. Biomed. Anal..

[20] Rasheed H, Höllein L, Holzgrabe U (2018). Future information technology tools for fighting substandard and falsified medicines in low- and middle-income countries. Front. Pharmacol..

[21] USP. <1850> Evaluation of screening technologies for assessing medicines quality. *USP 42-NRF 38* (2020).

[22] USP. *Technology Review Program*. https://www.usp.org/global-public-health/technology-review-program (Accessed 29 July 2022).

[23] Caillet C (2021). Evaluation of portable devices for medicine quality screening: Lessons learnt, recommendations for implementation, and future priorities. PLoS Med..

[24] Caillet C (2021). A comparative field evaluation of six medicine quality screening devices in Laos. PLoS Negl. Trop. Dis..

[25] Caillet C (2021). Multiphase evaluation of portable medicines quality screening devices. PLoS Negl. Trop. Dis..

[26] Luangasanatip N (2021). Implementation of field detection devices for antimalarial quality screening in Lao PDR-A cost-effectiveness analysis. PLoS Negl. Trop. Dis..

[27] Zambrzycki SC (2021). Laboratory evaluation of twelve portable devices for medicine quality screening. PLoS Negl. Trop. Dis..

[28] Hajjou M (2015). Monitoring the quality of medicines: Results from Africa, Asia, and South America. Am. J. Trop. Med. Hyg..

[29] Pribluda VS (2014). The three-level approach: A framework for ensuring medicines quality in limited-resource countries. Pharm. Reg. Aff..

[30] Global Pharma Health Fund e.V. *The GPHF-Minilab™*. https://www.gphf.org/en/index.htm (Accessed 29 July 2022).

[31] USP. USP Technology Review: Global Pharma Health Fund (GPHF) - Minilab™. Technology Review Program *Technology Review Program* (2020). https://www.usp.org/sites/default/files/usp/document/our-work/global-public-health/2020-usp-technology-review-global-pharma-health-fund-minilab.pdf (Accessed 29 July 2022).

[32] Pribluda VS (2012). Implementation of basic quality control tests for malaria medicines in Amazon Basin countries: Results for the 2005–2010 period. Malar. J..

[33] Risha P, Msuya Z, Ndomondo-Sigonda M, Layloff T (2006). Proficiency testing as a tool to assess the performance of visual TLC quantitation estimates. J. AOAC Int..

[34] WHO. Survey of the quality of selected antimalarial medicines circulating in six countries of sub-Saharan Africa. (2011). https://www.afro.who.int/sites/default/files/2017-06/WHO_QAMSA_report.pdf (Accessed 29 July 2022).

[35] Visser BJ (2015). Assessing the quality of anti-malarial drugs from Gabonese pharmacies using the MiniLab^®^: A field study. Malar. J..

[36] Schäfermann S (2020). Substandard and falsified antibiotics and medicines against noncommunicable diseases in western Cameroon and northeastern Democratic Republic of Congo. Am. J. Trop. Med. Hyg..

[37] Sherma J (2000). Planar chromatography. Anal. Chem..

[38] Sherma J, Rabel F (2019). Advances in the thin layer chromatographic analysis of counterfeit pharmaceutical products: 2008–2019. J. Liq. Chromatogr. Relat. Technol..

[39] Shewiyo DH (2012). HPTLC methods to assay active ingredients in pharmaceutical formulations: A review of the method development and validation steps. J. Pharm. Biomed. Anal..

[40] Tie-xin T, Hong W (2008). An image analysis system for thin-layer chromatography quantification and its validation. J. Chromatogr. Sci..

[41] Yu H (2016). Characterization of drug authenticity using thin-layer chromatography imaging with a mobile phone. J. Pharm. Biomed. Anal..

[42] Boulgakov AA (2020). Next-generation TLC: A quantitative platform for parallel spotting and imaging. J. Org. Chem..

[43] Gad A, Fayez Y, Kelani K, Mahmoud A (2021). TLC-smartphone in antibiotics determination and low-quality pharmaceuticals detection. RSC Adv..

[44] Tosato F (2016). Direct quantitative analysis of cocaine by thin layer chromatography plus a mobile phone and multivariate calibration: A cost-effective and rapid method. Anal. Methods.

[45] Biswas PC, Rani S, Hossain MA, Islam MR, Canning J (2022). Simultaneous multi-analyte sensing using a 2D quad-beam diffraction smartphone imaging spectrometer. Sens. Actuators B Chem..

[46] ICH. Validation of analytical procedures: text and methodology Q2(R1). ICH harmonised tripartite guidline. (2005). http://academy.gmp-compliance.org/guidemgr/files/Q2(R1).PDF (Accessed 29 July 2022).

[47] Rust Foundation. https://www.rust-lang.org (Accessed 29 July 2022).

[48] WHO. World Health Organization Model List of Essential Medicines—22nd List. (2021). https://apps.who.int/iris/bitstream/handle/10665/345533/WHO-MHP-HPS-EML-2021.02-eng.pdf.

[49] Jähnke, R.W.O. & Dwornik, K. *A Concise Quality Control Guide on Essential Drugs and Other Medicines. Physical Testing & Thin-Layer Chromatography. Review and Extensions 2020*, 3 ed. (Global Pharma Health Fund e. V. (GPHF), 2020).

[50] Hauk C, Hagen N, Heide L (2021). Identification of substandard and falsified medicines: Influence of different tolerance limits and use of authenticity inquiries. Am. J. Trop. Med. Hyg..

[51] TLCyzer. https://tlcyzer.github.io/ (Accessed 29 July 2022).

[52] UN. *Sustainable Development Goal 3: Ensure healthy lives and promote well-being for all at all ages*. https://sdgs.un.org/goals/goal3 (Accessed 29 July 2022).

[53] Kotlin Foundation. https://kotlinlang.org (Accessed 29 July 2022).

[54] Ballard DH (1981). Generalizing the Hough transform to detect arbitrary shapes. Pattern Recogn..

[55] Kacprowski, T. & Mehrtens, N. *linregress 0.4*, https://github.com/n1m3/linregress (2020).

