# Supplementary material for: An open-source smartphone app for the quantitative evaluation of thin-layer chromatographic analyses in medicine quality screening
Source: Sci Rep. 2022 Aug 4;12:13433. doi: 10.1038/s41598-022-17527-y (PMC9352711; doi:10.1038/s41598-022-17527-y)
Supplement: Supplementary file 1 — Supplementary Information. [file 41598_2022_17527_MOESM1_ESM.pdf]

Supplementary Information

An open-source smartphone app for the quantitative evaluation of thin-layer chromatographic analyses in medicine quality screening

Hauk C, Boss M, Gabel J, Schäfermann S, Lensch H, Heide L

Content	Page
Supplementary Figure S1: Spotting patterns on the TLC plate, and different croppings of the TLC photo	2
Supplementary Figure S2: Construction plan of the box for photography of TLC plates under UV illumination	3-5
Supplementary Figure S3: Step-by-step instructions for the use of the TLCyzer app	6-9
Supplementary Figure S4: Exemplary photos of TLC analysis of the 14 investigated APIs	10
Supplementary Figure S5: Boxes for photography of TLC plates, reproduced by (a) a workshop in Germany and (b) by a carpenter in Zimbabwe	11
Supplementary Table S1: Composition of the TLC mobile phases for the 14 investigated APIs according to the GPHF Minilab manual	12
Supplementary Table S2: Results of individual measurements for the evaluation of accuracy and repeatability	13
Supplementary Table S3: Results of individual measurements for the evaluation of intermediate precision	14-16
Supplementary Table S4: Results of individual measurements for the evaluation of linearity	17
Supplementary Table S5: Results of individual measurements for the evaluation of robustness	18
Supplementary Table S6: Results of TLCyzer analysis of four finished pharmaceutical products of good quality, and of two substandard pharmaceutical products	19
Supplementary Table S7: Results of TLCyzer analysis using 80% and 100% reference solutions only	20

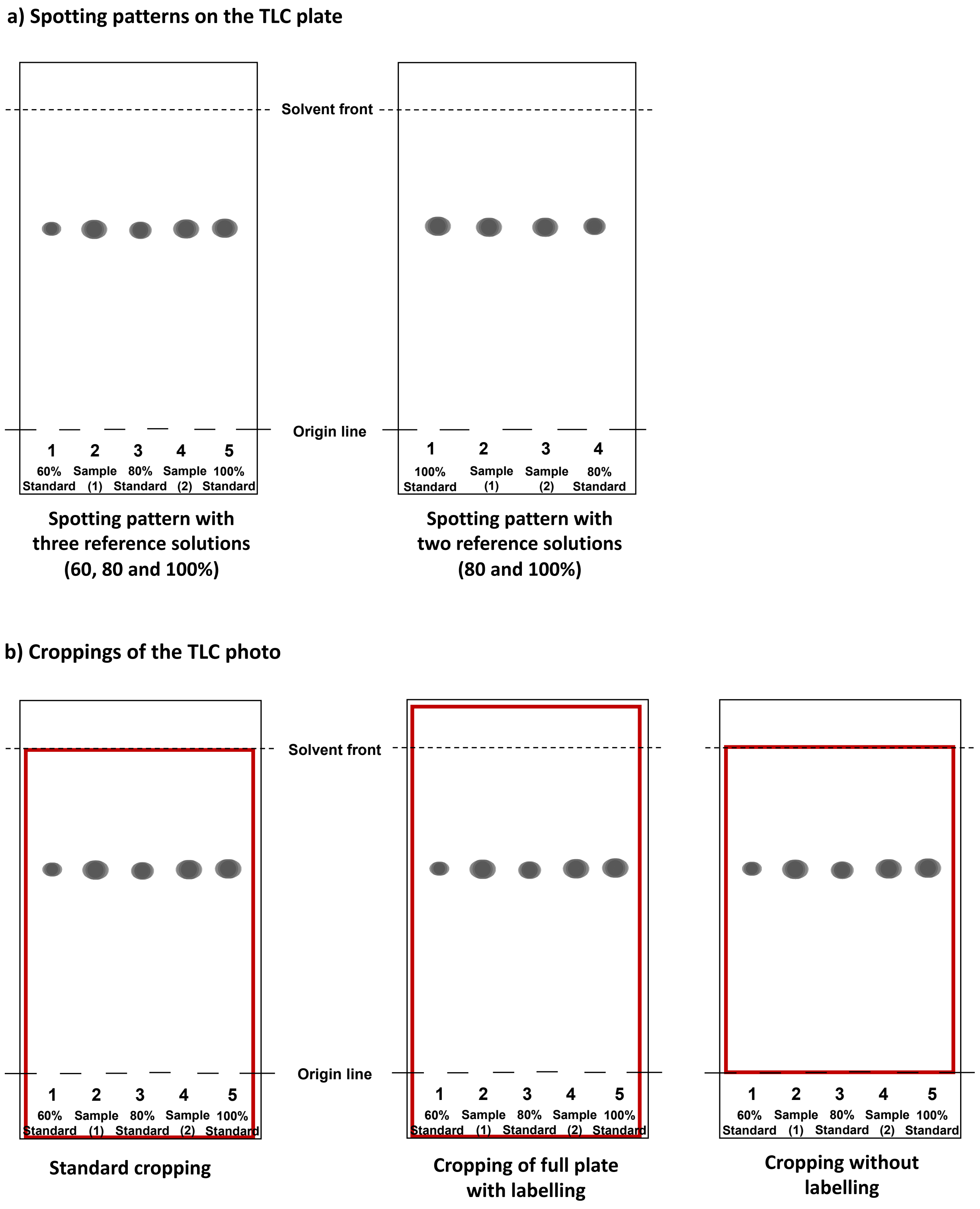

Supplementary Figure S1: a) Spotting patterns on the TLC plate. The spotting pattern with three reference solutions was used in the evaluation, unless indicated otherwise. **b)** Different croppings of the TLC photo, used in the evaluation of robustness. The evaluated section of the photo is highlighted in red.

a) Assembled box

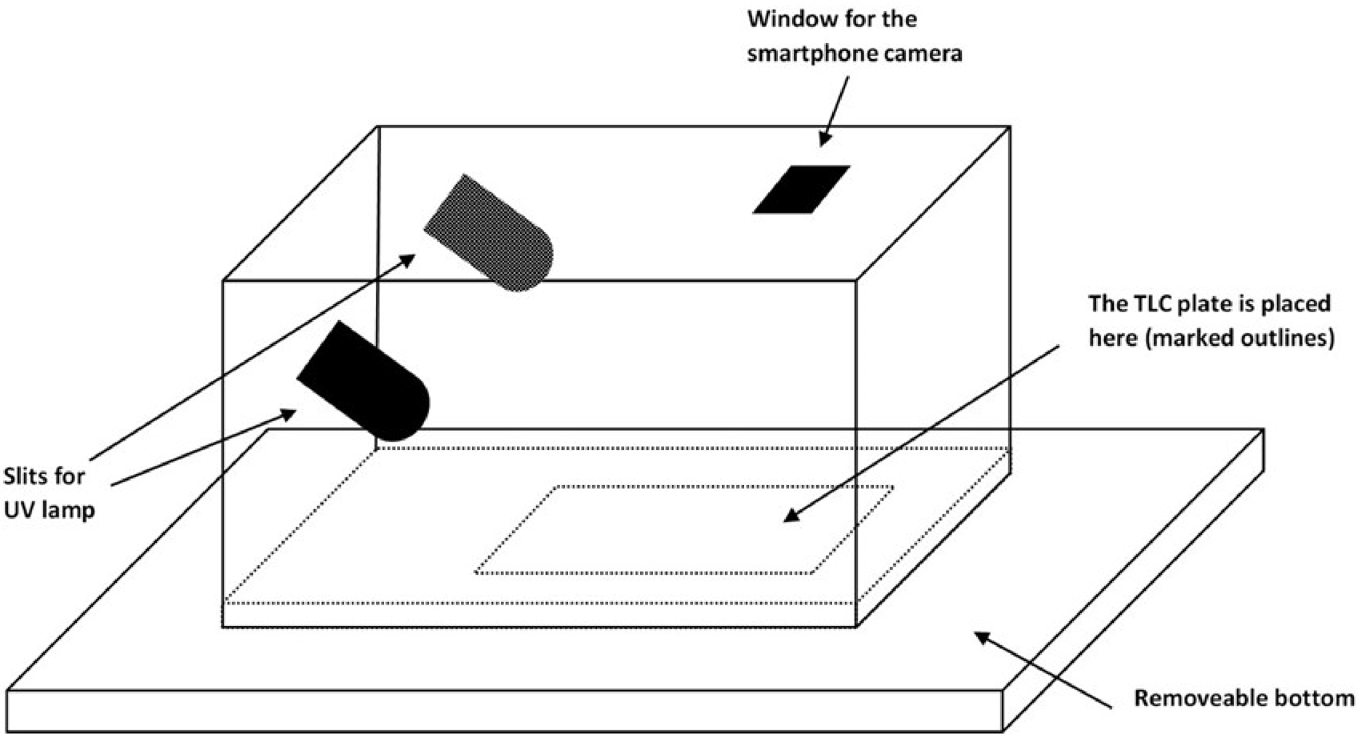

b) Bottom of the box

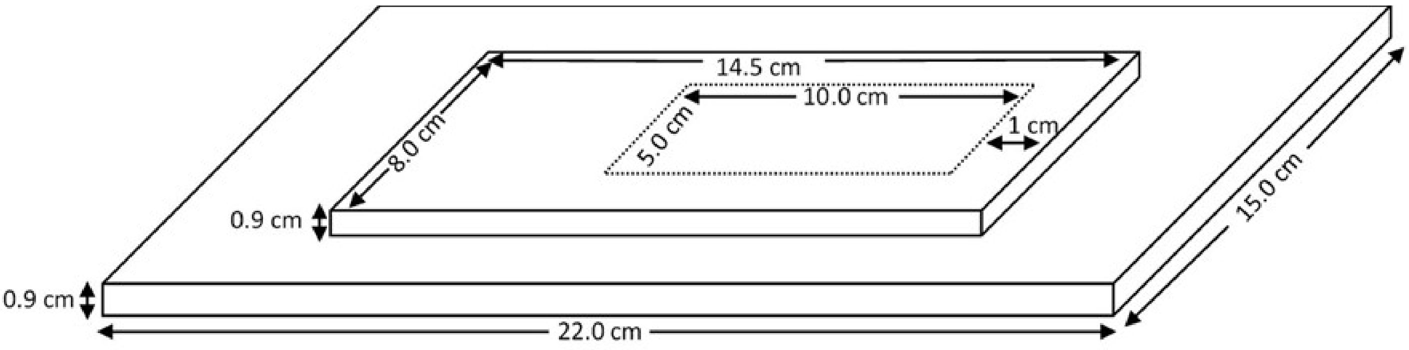

c) Lid of the box

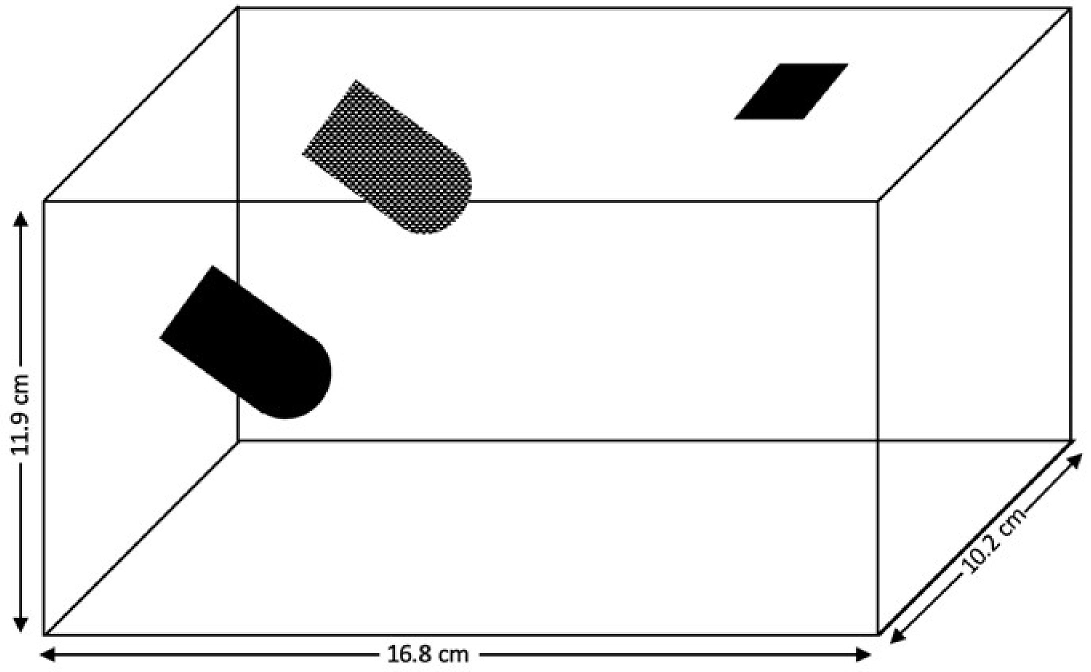

d) Side view of the lid

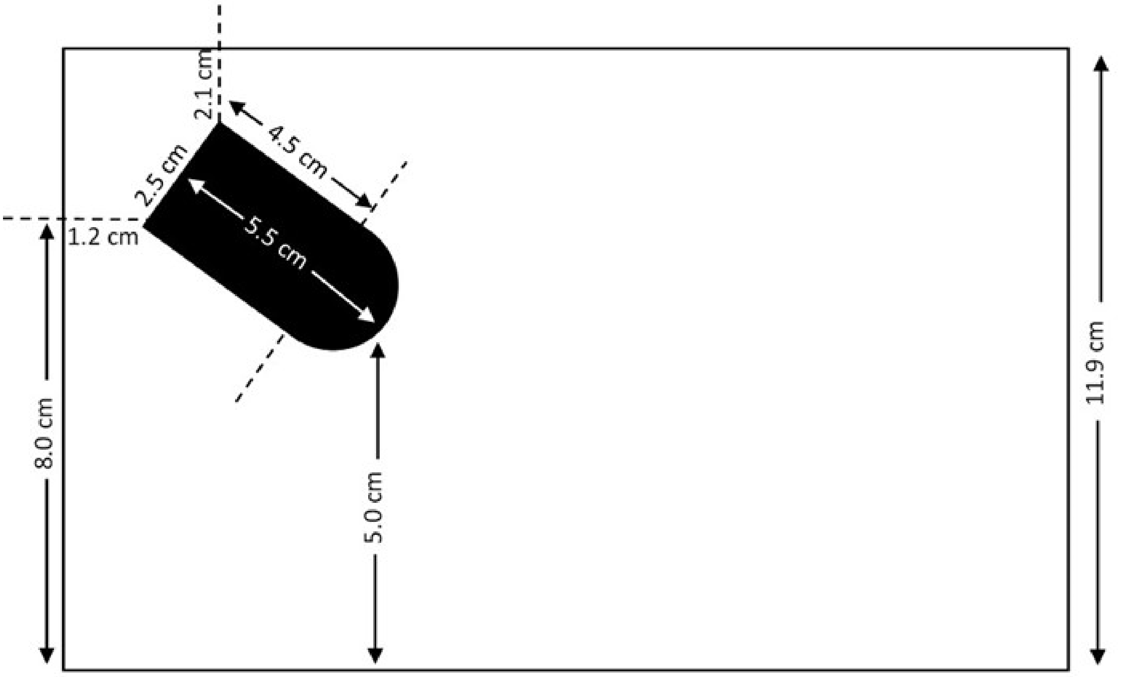

e) Top view on the box

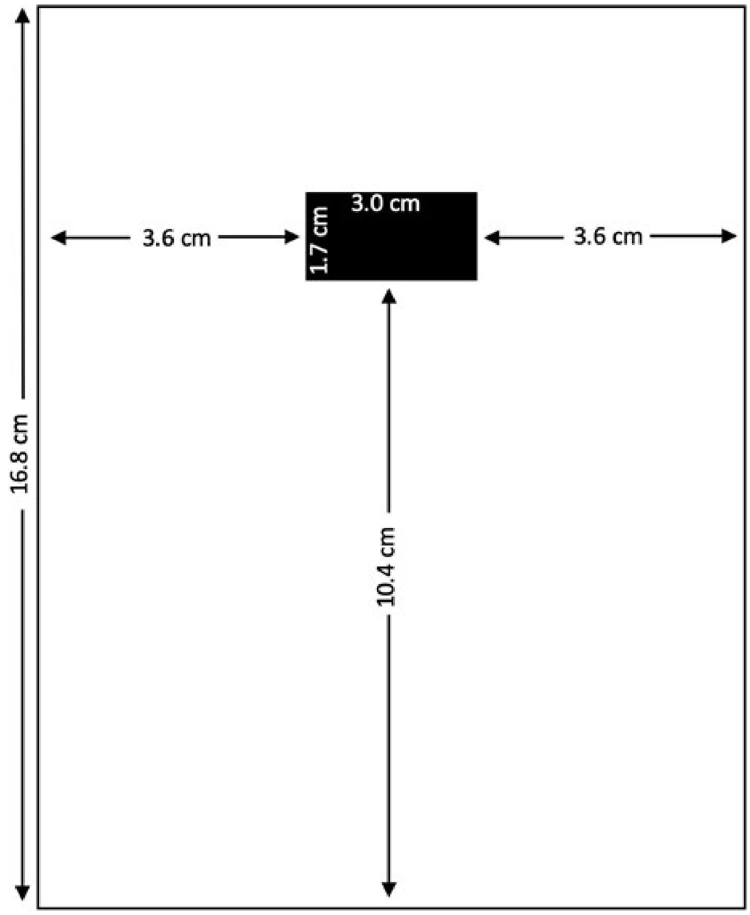

Supplementary Figure S2: Construction plan of the box for photography of TLC plates under UV illumination.
a) The box consists of a removeable lid and a bottom plate. The smartphone is placed on the lid of the box, with the camera facing the TLC plate through the opening in the lid. **b)** Bottom of the box. The TLC plate is to be placed on a marked 5 x 10 cm rectangle on the raised 8 x 14.5 cm platform. The raised platform is firmly glued to the 15 x 22 cm lower part of the bottom. **c)** Lid of the box. **d)** Side view of the lid, showing the position of the openings for the UV lamp. **e)** Top view of the box, showing the position of the opening for the smartphone camera.

The box is coloured on the in- and outside with a black, matte colour to minimize reflections. It consists of wooden plates of 9 mm thickness. Plates with a different thickness may be used, but in this case the dimensions of the box must be adjusted accordingly.

Instructions for the quantitative evaluation of TLC plates (5 x 10 cm) with the TLCyzer app

What you need:

- Developed and dry TLC plate
- UV lamp 254nm
- Box for photographing TLC plates (Figure S1)
- Smartphone with a rear facing camera (Android operating system)
- Internet connection to download the TLCyzer app

Before you start:

- Search for "TLCyzer app" in the Google Play Store and install it on the smartphone
- Put the developed TLC plate into the marked rectangle on the bottom of the box, and assemble the box by placing the lid over the bottom
- Insert the UV lamp, and place it centrally in the box
- Switch on the UV lamp and start the TLCyzer app on your smartphone

1

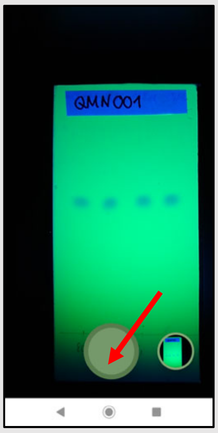

2

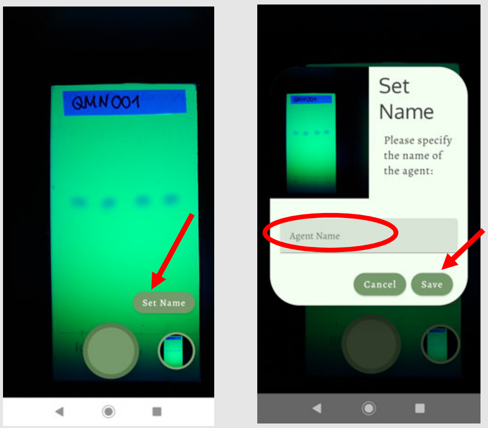

1. Photo of the TLC plate

After starting the app, the smartphone camera turns on automatically. Take a photo of the TLC plate by tapping on the large circle.

2. Name of the sample

Tap on "Set Name", enter a suitable sample name and tap on "Save".

3

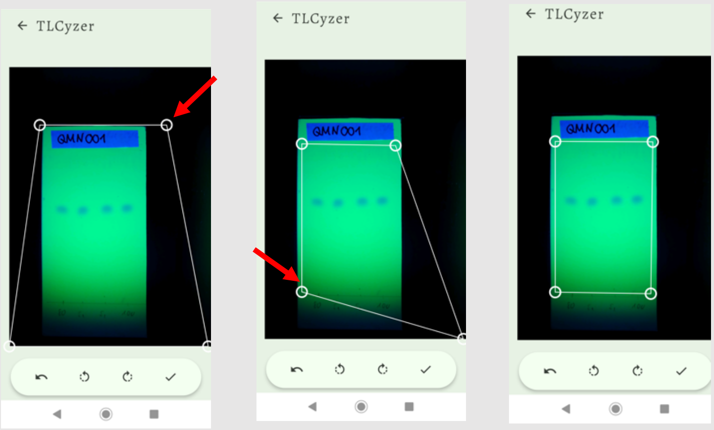

3. TLC plate recognition

If the outlines of the plate are not correctly recognized by the app, you have to set them manually.

The four adjustable corner points can be used to set the correct outlines of the plate.

Make sure that the selected square is rectangular and only contains the plate and no parts of the black background, and no parts of any labels which you may have attached to the plate.

4

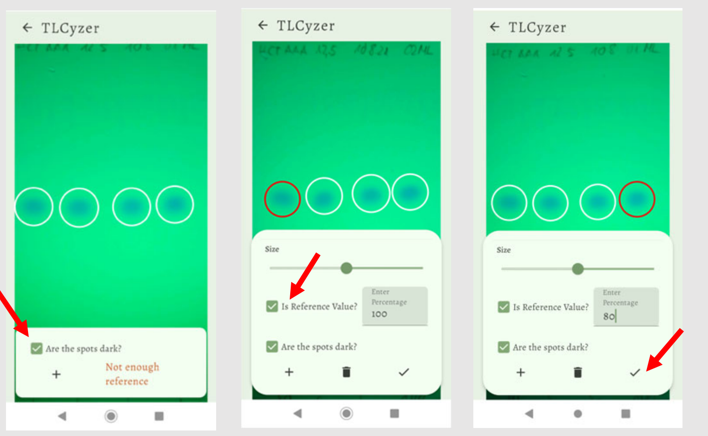

4. Spot detection

The spots are automatically detected by the app, indicated by the white circles around the spots. Please leave the tick at “Are the spots dark?”.

To mark a spot as a reference with known concentration, tap on it (it turns red) and place a tick at “Is Reference Value”. Then enter the respective concentration in the field “Enter percentage” (e.g. 100% and 80%).

Tap on the tick at the bottom right to start the evaluation.

5

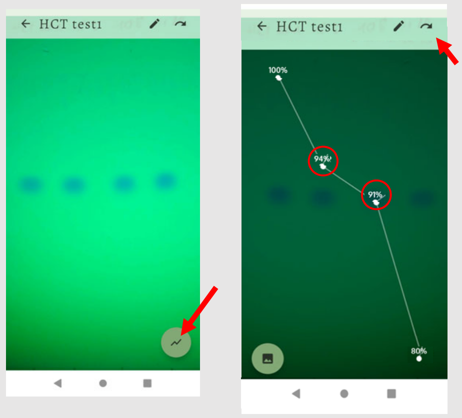

5. Result

The result for the reference and sample spots is now displayed on the smartphone screen. Calculate the mean of the two sample spots. This is your analysis result.

You can repeat the analysis by tapping at the arrow at the top right corner. The previously chosen outlines of the plate, and reference concentrations, are displayed, preset and can be adjusted if necessary.

By tapping at the pencil at the upper right corner you can (re)name the photo.

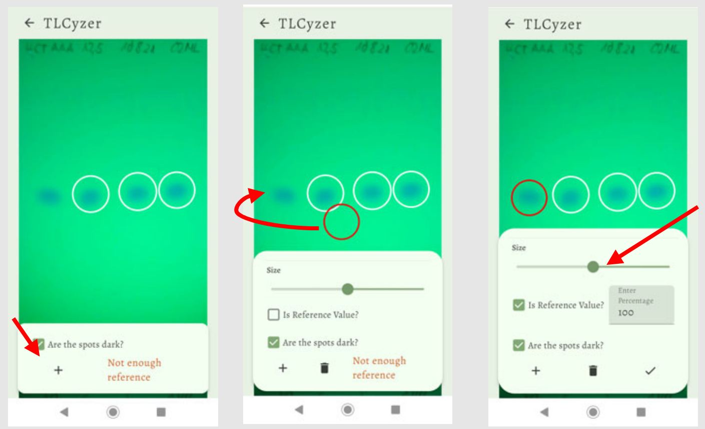

How to add circles

If some or all the spots have not been detected automatically, circles can be added manually. Tap on the + symbol at the bottom left and a circle appears. Draw the circle over the spot, making sure that the spot is placed centrally in the circle. The size of the circle can be adjusted using the slide.

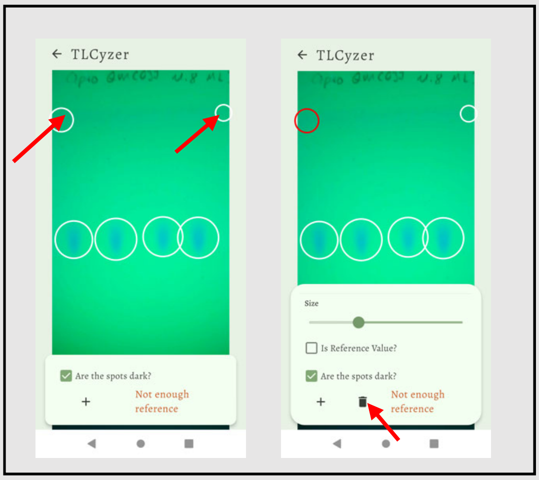

How to delete circles

In addition to the spots of the active pharmaceutical ingredient, additional circles may sometime appear, resulting from markings or contaminations of the TLC plate. These additional circles must be deleted. To do this, tap on the circle (it turns red) and tap on the waste bin symbol.

Note: If you analyse a combination product that contains two APIs, you can only evaluate one API per analysis. The circles around the spots of the other API have to be deleted. Take another photo of the TLC plate to evaluate the second API.

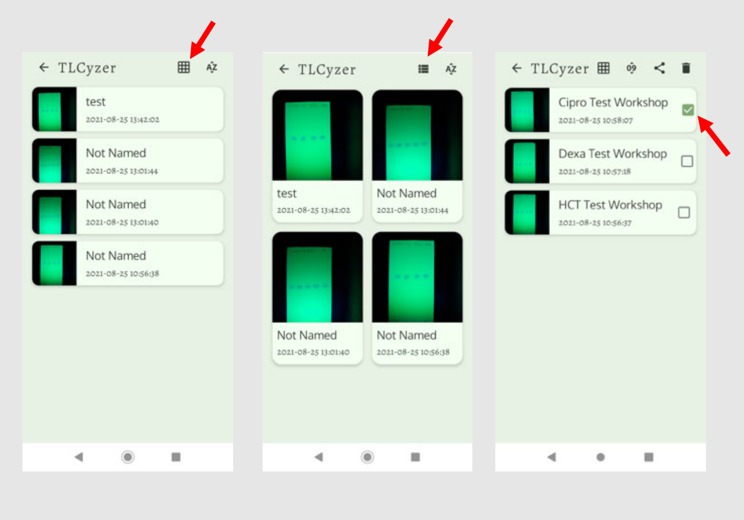

Photo gallery / How to select and delete photos

All photos are stored within the app and can be sorted by date, and alphabetically by name. In the photo gallery, it is possible to switch between grid and list view.

Photos can be selected by tapping on them for two seconds (a tick appears on the right).

This is necessary if you want to share a photo (see next step) or if you want to delete it. To delete photo(s), select them and tap at the waste bin symbol.

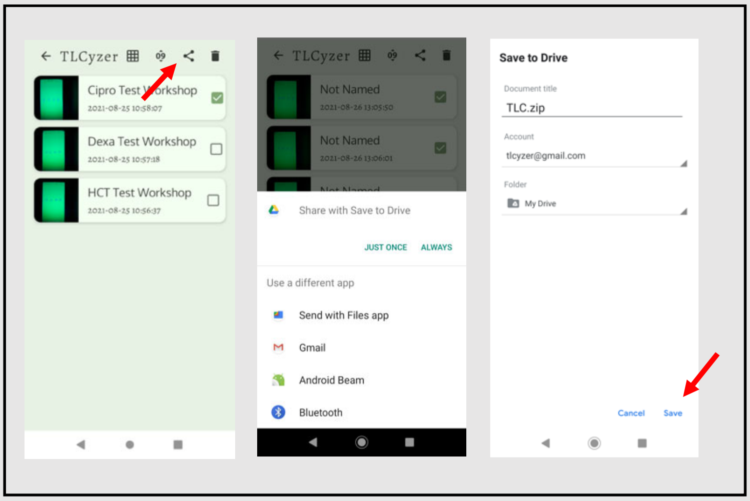

How to share or save photos online

Select the photo(s) you want share (see above) and tap at the share icon that appears in the top bar of the screen. Then a new window appears at the bottom. You can now choose how you want to share the photo(s) e.g. by WhatsApp, e-mail, Bluetooth, or by uploading them to a cloud storage service; if you choose e.g. Google Drive (see photo on the left) enter the "Document title", your "Account" (e-mail address) and tap on "Save" at the bottom right. The photo(s) are now saved as a ZIP file in your Google Drive account.

<u>How to download photos to a computer using a cable (rather than wireless connection)</u>
Select the photo(s) you want to download and click at the share icon. This will create a ZIP file containing these photos. Connect the smartphone to the computer with an appropriate cable. You will find all the photos and the created ZIP file if you open the following folders of the smartphone on the computer: “Android”, “data”, “de.uni.tuebingen.tlceval”, “files”, “TLCyzer”.

<u>How to import photos into the app</u>
If you want to evaluate a TLC photo taken with the app on another smartphone, it must first be imported into the app of the smartphone used via the share function. If necessary, unzip the file and then download the photo(s) to your smartphone. Select the photo(s) and import them into the TLCyzer app using the share function. The photo(s) are then available in the gallery of the TLCyzer app and can be evaluated.

Supplementary Figure S3: Step-by-step instructions for the use of the TLCyzer app (version 0.3).

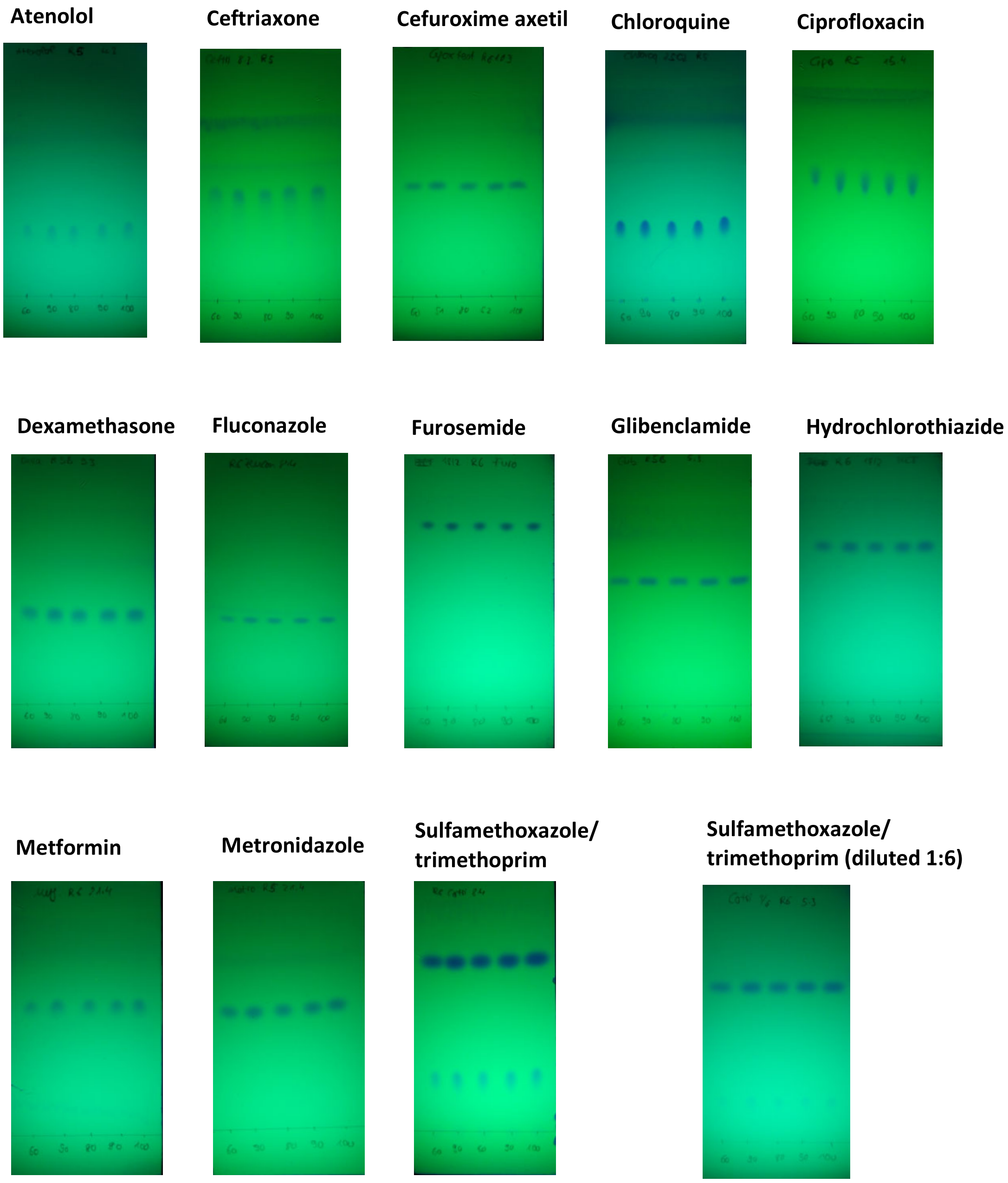

Supplementary Figure S4: Exemplary photos of TLC analysis of the 14 investigated APIs.
For each API, a sample solution containing 90% of the standard concentration given in Table 1 was applied to lanes 2 and 4 of the TLC plates. Solutions of reference substances containing 60%, 80% and 100% of the standard concentrations (given in Table 1) were applied to lanes 1, 3 and 5, respectively. Plates were developed and photographed as explained in the Methods section. Sulfamethoxazole and trimethoprim were combined in the respective solutions, in accordance with their fixed combinations in cotrimoxazole tablets. For the last photo, test and reference solutions of sulfamethoxazole/trimethoprim were diluted by a factor of six (see Methods).

a

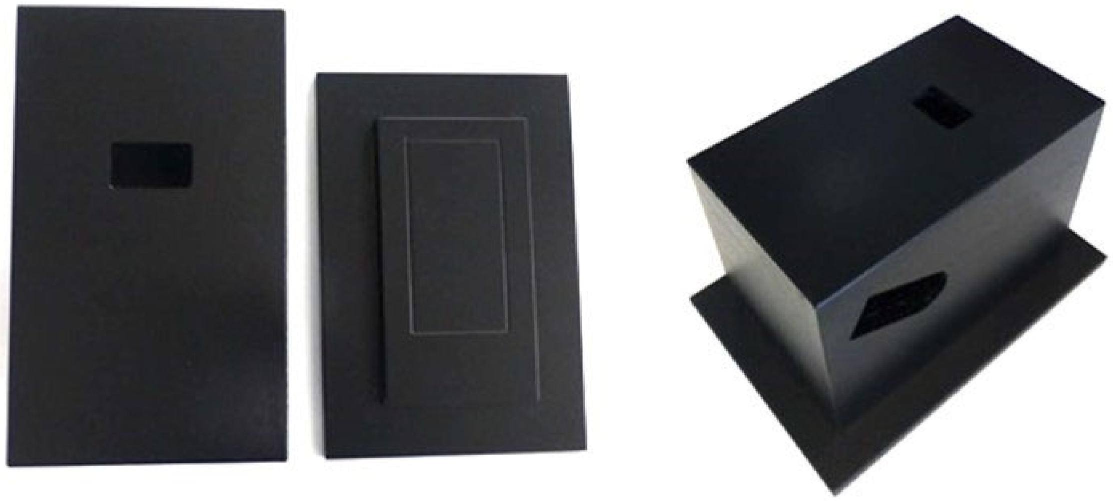

b

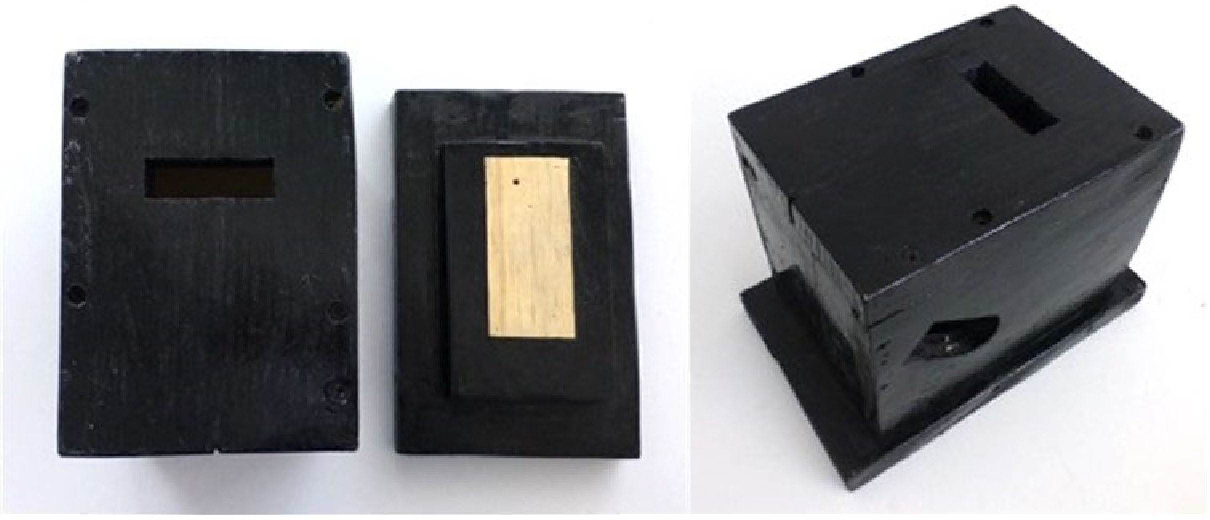

Supplementary Figure S5: Boxes for photography of TLC plates reproduced by (a) a workshop in Germany (Pidinger Werkstätten, Piding, Germany); (b) a carpenter in Zimbabwe (see Methods). The box shown in (a) is glued with a waterproof adhesive, the one in (b) is screwed. Both methods are fine.

API	Composition of the mobile phase in the development chamber (based on 20 ml)							
	Methanol	Acetone	Water	Aqueous ammonia solution (25%)	Acetic acid (96%)	Ethyl acetate	Toluene	Magnesium chloride hexahydrate
**Atenolol**	20 ml			0.2 ml				
**Ceftriaxone sodium in powder for injections**	6 ml	4 ml	2 ml			8 ml		1 g
**Cefuroxime axetil**		10 ml			1 ml		10 ml	
**Chloroquine phosphate**	20 ml			0.5 ml		5 ml		
**Ciprofloxacin HCl**	10 ml	5 ml		5 ml			2.5 ml	
**Dexamethasone**		2 ml	0.2 ml			20 ml		
**Fluconazole**	6 ml			0.5 ml			14 ml	
**Furosemide**	15 ml				1 ml		5 ml	
**Glibenclamide**	7 ml			1 ml		11 ml	1 ml	
**Hydrochlorothiazide**	2 ml				1 ml	17 ml		
**Metformin HCl**	15 ml		5 ml		1 ml			
**Metronidazole**	5 ml			10 drops		15 ml		
**Cotrimoxazole** (sulfamethoxazole and trimethoprim)	5 ml					15 ml		

Supplementary Table S1: Composition of the TLC mobile phases for the 14 investigated APIs according to the GPHF Minilab manual

API	Conc. [%]	Result (accuracy) [%]						Recovery [%]				
		TLC plate 1		TLC plate2		Mean	RSD	TLC plate 1		TLC plate2		Mean
		left spot	right spot	left spot	right spot			left spot	right spot	left spot	right spot	
Atenolol	70	72.6	72.3	73.2	71.8	72.5	**0.81**	103.7	103.3	104.6	102.6	**103.5**
	85	85.9	87.6	86.4	83.8	85.9	**1.85**	101.1	103.1	101.6	98.6	**101.1**
	90	91.9	94.9	86.0	93.8	91.7	**4.33**	102.1	105.4	95.6	104.2	**101.8**
Ceftriaxone	70	75.5	69.9	69.2	65.9	70.1	**5.68**	107.9	99.9	98.9	94.1	**100.2**
	85	83.8	84.3	85.1	86.1	84.8	**1.18**	98.6	99.2	100.1	101.3	**99.8**
	90	93.3	88.3	94.7	88.4	91.2	**3.63**	103.7	98.1	105.2	98.2	**101.3**
Cefuroxime axetil	70	71.2	73.9	74.9	68.0	72.0	**4.29**	101.7	105.6	107.0	97.1	**102.9**
	85	80.1	82.0	85.2	81.8	82.3	**2.59**	94.2	96.5	100.2	96.2	**96.8**
	90	93.7	85.8	93.5	88.9	90.5	**4.23**	104.1	95.3	103.9	98.8	**100.5**
Chloroquine	70	69.3	67.9	67.8	69.6	68.7	**1.36**	99.0	97.0	96.9	99.4	**98.1**
	85	85.0	84.5	85.6	86.7	85.5	**1.11**	100.0	99.4	100.7	102.0	**100.5**
	90	89.8	91.2	94.8	91.7	91.9	**2.30**	99.8	101.3	105.3	101.9	**102.1**
Ciprofloxacin	70	70.0	67.7	69.1	72.3	69.8	**2.77**	100.0	96.7	98.7	103.3	**99.7**
	85	85.4	81.6	89.4	82.4	84.7	**4.17**	100.5	96.0	105.2	96.9	**99.6**
	90	95.0	90.1	89.9	94.9	92.5	**3.09**	105.6	100.1	99.9	105.4	**102.8**
Dexamethasone	70	71.6	66.4	65.7	66.3	67.5	**4.08**	102.3	94.9	93.9	94.7	**96.4**
	85	85.1	83.0	88.0	84.8	85.2	**2.43**	100.1	97.6	103.5	99.8	**100.3**
	90	92.6	90.5	90.4	94.8	92.1	**2.26**	102.9	100.6	100.4	105.3	**102.3**
Fluconazole	70	70.2	71.7	71.6	68.1	70.4	**2.39**	100.3	102.4	102.3	97.3	**100.6**
	85	86.5	83.9	83.5	83.1	84.3	**1.82**	101.8	98.7	98.2	97.8	**99.1**
	90	89.7	91.6	85.3	89.8	89.1	**3.01**	99.7	101.8	94.8	99.8	**99.0**
Furosemide	70	73.0	68.9	69.8	69.6	70.3	**2.59**	104.3	98.4	99.7	99.4	**100.5**
	85	85.7	87.9	83.9	82.9	85.1	**2.58**	100.8	103.4	98.7	97.5	**100.1**
	90	90.8	91.8	93.4	94.2	92.6	**1.66**	100.9	102.0	103.8	104.7	**102.8**
Glibenclamide	70	70.1	70.4	67.1	66.4	68.5	**2.98**	100.1	100.6	95.9	94.9	**97.9**
	85	80.0	80.0	89.2	82.7	83.0	**5.23**	94.1	94.1	104.9	97.3	**97.6**
	90	90.2	92.1	89.3	90.6	90.6	**1.29**	100.2	102.3	99.2	100.7	**100.6**
Hydrochloro-thiazide	70	70.2	72.7	70.5	69.9	70.8	**1.80**	100.3	103.9	100.7	99.9	**101.2**
	85	86.5	84.9	86.5	84.0	85.5	**1.45**	101.8	99.9	101.8	98.8	**100.6**
	90	91.1	90.6	93.9	87.9	90.9	**2.70**	101.2	100.7	104.3	97.7	**101.0**
Metformin	70	72.6	66.9	73.9	74.4	72.0	**4.80**	103.7	95.6	105.6	106.3	**102.8**
	85	82.7	81.5	85.4	85.3	83.7	**2.32**	97.3	95.9	100.5	100.4	**98.5**
	90	91.7	88.7	89.8	86.3	89.1	**2.53**	101.9	98.6	99.8	95.9	**99.0**
Metronidazole	70	70.5	67.4	66.9	71.8	69.2	**3.44**	100.7	96.3	95.6	102.6	**98.8**
	85	85.2	84.0	85.4	85.3	85.0	**0.77**	100.2	98.8	100.5	100.4	**100.0**
	90	88.4	87.4	92.4	91.9	90.0	**2.77**	98.2	97.1	102.7	102.1	**100.0**
Sulfa-methoxazole	70	73.4	65.4	70.6	71.1	70.1	**4.82**	104.9	93.4	100.9	101.6	**100.2**
	85	86.7	83.8	88.4	90.7	87.4	**3.33**	102.0	98.6	104.0	106.7	**102.8**
	90	97.0	93.9	89.3	93.9	93.5	**3.39**	107.8	104.3	99.2	104.3	**103.9**
Trimethoprim	70	70.4	67.1	69.3	67.6	68.6	**2.22**	100.6	95.9	99.0	96.6	**98.0**
	85	83.0	81.5	83.3	88.1	84.0	**3.41**	97.6	95.9	98.0	103.6	**98.8**
	90	86.2	87.6	89.9	88.9	88.2	**1.82**	95.8	97.3	99.9	98.8	**97.9**
**Mean**							**2.79**					**100.3**

Supplementary Table S2: Results of individual measurements for the evaluation of accuracy and repeatability.

API	Operator	Photo No.	Analysis day	Result [%] (true value 90%)					Recovery [%]	
				left spot	right spot	Mean	SD	RSD	left spot	right spot
**Atenolol**	Y.W.	1	1	88.2	88.8	88.5	4.38	4.94	98.0	98.7
	C.H.	1	1	83.2	91.4				92.4	101.6
	Y.W.	2	1	80.7	90.0				89.7	100.0
	J.G.	2	1	88.7	88.3				98.6	98.1
	C.H.	2	1	85.1	88.0				94.6	97.8
	J.G.	3	1	89.8	94.4				99.8	104.9
	J.G.	1	2	88.9	91.3				98.8	101.4
	C.H.	1	2	88.4	92.8				98.2	103.1
	J.G.	2	2	86.6	83.6				96.2	92.9
	C.H.	2	2	82.1	84.6				91.2	94.0
	Y.W.	3	2	89.5	100.9				99.4	112.1
	C.H.	3	2	86.2	93.2				95.8	103.6
**Ceftriaxone**	Y.W.	1	1	89.3	89.0	88.1	2.82	3.20	99.2	98.9
	J.G.	1	1	87.4	84.1				97.1	93.4
	C.H.	1	1	89.8	88.0				99.8	97.8
	J.G.	2	1	91.1	86.8				101.2	96.4
	C.H.	2	1	95.3	88.8				105.9	98.7
	Y.W.	3	1	92.4	87.7				102.7	97.4
	Y.W.	1	2	89.5	90.3				99.4	100.3
	C.H.	1	2	87.0	84.1				96.7	93.4
	J.G.	2	2	87.9	85.7				97.7	95.2
	Y.W.	3	2	90.8	86.7				100.9	96.3
	J.G.	3	2	84.9	83.5				94.3	92.8
	C.H.	3	2	88.9	85.2				98.8	94.7
**Cefuroxime axetil**	J.G.	1	1	94.7	89.7	91.2	4.57	5.01	105.2	99.7
	C.H.	1	1	83.5	82.7				92.8	91.9
	J.G.	2	1	97.4	88.5				108.2	98.3
	C.H.	2	1	87.8	87.5				97.6	97.2
	Y.W.	3	1	96.5	89.6				107.2	99.6
	C.H.	3	1	87.8	87.5				97.6	97.2
	J.G.	1	2	94.7	89.7				105.2	99.7
	C.H.	1	2	95.1	89.8				105.7	99.8
	Y.W.	2	2	93.7	90.0				104.1	100.0
	J.G.	2	2	98.7	88.6				109.7	98.4
	Y.W.	3	2	93.8	87.6				104.2	97.3
	J.G.	3	2	100.3	92.9				111.4	103.2
**Chloroquine**	Y.W.	1	1	93.1	99.2	91.8	3.67	4.00	103.4	110.2
	C.H.	1	1	85.8	89.8				95.3	99.8
	J.G.	2	1	88.2	91.5				98.0	101.7
	Y.W.	3	1	94.3	93.5				104.8	103.9
	J.G.	3	1	85.2	90.6				94.7	100.7
	C.H.	3	1	92.0	94.4				102.2	104.9
	J.G.	1	2	100.3	92.9				111.4	103.2
	C.H.	1	2	86.7	90.7				96.3	100.8
	Y.W.	2	2	89.0	97.0				98.9	107.8
	J.G.	2	2	91.3	92.2				101.4	102.4
	C.H.	2	2	90.1	91.3				100.1	101.4
	C.H.	3	2	92.3	91.1				102.6	101.2
**Ciprofloxacin**	Y.W.	1	1	94.0	86.6	90.5	4.55	5.03	104.4	96.2
	J.G.	1	1	90.6	85.3				100.7	94.8
	C.H.	1	1	93.7	87.1				104.1	96.8
	C.H.	2	1	95.7	86.3				106.3	95.9
	Y.W.	3	1	93.5	90.0				103.9	100.0
	J.G.	3	1	92.2	85.7				102.4	95.2
	Y.W.	1	2	93.1	87.6				103.4	97.3
	C.H.	1	2	90.5	87.9				100.6	97.7
	Y.W.	2	2	94.8	86.3				105.3	95.9
	C.H.	2	2	96.0	91.8				106.7	102.0

API	Operator	Photo No.	Analysis day	Result [%] (true value 90%)					Recovery [%]	
				left spot	right spot	Mean	SD	RSD	left spot	right spot
	J.G.	3	2	101.5	80.7				112.8	89.7
	C.H.	3	2	92.9	87.8				103.2	97.6
**Dexametha-sone**	Y.W.	1	1	94.3	87.5	90.8	4.04	4.45	104.8	97.2
	J.G.	1	1	91.0	87.7				101.1	97.4
	C.H.	1	1	93.5	88.7				103.9	98.6
	Y.W.	2	1	96.4	88.9				107.1	98.8
	J.G.	2	1	99.1	88.0				110.1	97.8
	Y.W.	3	1	91.7	90.3				101.9	100.3
	J.G.	1	2	99.8	88.9				110.9	98.8
	C.H.	1	2	94.5	87.0				105.0	96.7
	Y.W.	2	2	92.5	89.8				102.8	99.8
	C.H.	2	2	90.2	86.8				100.2	96.4
	J.G.	3	2	83.8	86.3				93.1	95.9
	C.H.	3	2	93.6	88.0				104.0	97.8
**Fluconazole**	Y.W.	1	1	90.7	92.5	89.6	3.22	3.60	100.8	102.8
	C.H.	1	1	90.9	93.6				101.0	104.0
	J.G.	2	1	86.6	85.6				96.2	95.1
	Y.W.	3	1	87.8	90.1				97.6	100.1
	J.G.	3	1	99.9	89.3				111.0	99.2
	C.H.	3	1	82.5	86.5				91.7	96.1
	J.G.	1	2	87.7	90.1				97.4	100.1
	C.H.	1	2	88.3	88.9				98.1	98.8
	Y.W.	2	2	89.1	91.4				99.0	101.6
	J.G.	2	2	90.0	90.7				100.0	100.8
	C.H.	2	2	90.8	88.9				100.9	98.8
	Y.W.	3	2	87.7	90.0				97.4	100.0
**Furosemide**	J.G.	1	1	85.0	90.0	91.1	3.58	3.93	94.4	100.0
	C.H.	1	1	90.3	87.3				100.3	97.0
	J.G.	2	1	88.4	94.4				98.2	104.9
	Y.W.	3	1	95.8	97.1				106.4	107.9
	J.G.	3	1	87.8	89.8				97.6	99.8
	C.H.	3	1	95.0	89.4				105.6	99.3
	Y.W.	1	2	97.1	93.2				107.9	103.6
	C.H.	1	2	88.6	87.8				98.4	97.6
	Y.W.	2	2	85.5	92.8				95.0	103.1
	J.G.	2	2	88.4	91.0				98.2	101.1
	C.H.	2	2	90.9	89.4				101.0	99.3
	Y.W.	3	2	94.7	95.5				105.2	106.1
**Glibenclamide**	Y.W.	1	1	89.2	92.9	90.5	2.87	3.17	99.1	103.2
	C.H.	1	1	89.7	93.7				99.7	104.1
	Y.W.	2	1	88.4	93.1				98.2	103.4
	J.G.	2	1	88.4	92.0				98.2	102.2
	C.H.	2	1	88.7	92.7				98.6	103.0
	J.G.	3	1	89.6	93.4				99.6	103.8
	J.G.	1	2	91.0	92.4				101.1	102.7
	C.H.	1	2	88.7	92.5				98.6	102.8
	Y.W.	2	2	88.7	93.6				98.6	104.0
	C.H.	2	2	86.4	85.4				96.0	94.9
	Y.W.	3	2	86.3	95.7				95.9	106.3
	J.G.	3	2	86.5	93.4				96.1	103.8
**Hydrochloro-thiazide**	Y.W.	1	1	91.3	84.5	90.4	4.07	4.50	101.4	93.9
	C.H.	1	1	94.7	90.3				105.2	100.3
	J.G.	2	1	91.6	88.4				101.8	98.2
	Y.W.	3	1	97.7	88.8				108.6	98.7
	J.G.	3	1	84.7	92.5				94.1	102.8
	C.H.	3	1	92.8	85.4				103.1	94.9
	J.G.	1	2	88.5	88.8				98.3	98.7
	C.H.	1	2	88.8	89.9				98.7	99.9

API	Operator	Photo No.	Analysis day	Result [%] (true value 90%) left spot	right spot	Mean	SD	RSD	Recovery [%] left spot	right spot
	Y.W.	2	**2**	95.1	92.3				105.7	102.6
	J.G.	2	**2**	81.3	87.5				90.3	97.2
	C.H.	2	**2**	95.6	89.3				106.2	99.2
	Y.W.	3	**2**	93.8	96.0				104.2	106.7
**Metformin**	Y.W.	1	1	88.5	89.1	89.1	3.70	4.15	98.3	99.0
	C.H.	1	1	92.4	89.1				102.7	99.0
	J.G.	2	1	92.9	83.9				103.2	93.2
	Y.W.	3	1	88.2	87.5				98.0	97.2
	J.G.	3	1	96.7	97.1				107.4	107.9
	C.H.	3	1	91.6	87.2				101.8	96.9
	Y.W.	1	2	88.0	87.7				97.8	97.4
	C.H.	1	2	89.4	86.5				99.3	96.1
	Y.W.	2	2	86.3	87.3				95.9	97.0
	J.G.	2	2	90.1	83.8				100.1	93.1
	C.H.	2	2	86.7	87.4				96.3	97.1
	J.G.	3	2	96.4	85.2				107.1	94.7
**Metronida-zole**	J.G.	1	1	95.1	93.4	91.6	4.32	4.71	105.7	103.8
	C.H.	1	1	90.3	86.2				100.3	95.8
	Y.W.	2	1	100.8	88.4				112.0	98.2
	J.G.	2	1	95.6	91.4				106.2	101.6
	C.H.	2	1	91.9	87.2				102.1	96.9
	C.H.	3	1	83.5	90.3				92.8	100.3
	Y.W.	1	2	88.3	85.7				98.1	95.2
	C.H.	1	2	91.8	92.5				102.0	102.8
	Y.W.	2	2	102.3	92.3				113.7	102.6
	J.G.	2	2	89.9	92.5				99.9	102.8
	J.G.	3	2	95.6	92.3				106.2	102.6
	C.H.	3	2	89.9	92.3				99.9	102.6
**Sulfamethox-azole**	Y.W.	1	1	87.9	93.5	88.3	7.08	8.02	97.7	103.9
	J.G.	1	1	76.4	76.8				84.9	85.3
	Y.W.	2	1	93.0	91.4				103.3	101.6
	J.G.	2	1	79.1	99.3				87.9	110.3
	C.H.	2	1	91.0	93.3				101.1	103.7
	J.G.	3	1	79.6	88.8				88.4	98.7
	J.G.	1	2	94.9	95.8				105.4	106.4
	C.H.	1	2	80.8	83.5				89.8	92.8
	Y.W.	2	2	92.4	97.4				102.7	108.2
	C.H.	2	2	86.7	100.1				96.3	111.2
	Y.W.	3	2	86.3	87.4				95.9	97.1
	C.H.	3	2	82.9	81.1				92.1	90.1
**Trimethoprim**	Y.W.	1	1	89.7	95.6	91.9	3.38	3.68	99.7	106.2
	J.G.	1	1	88.5	89.4				98.3	99.3
	Y.W.	2	1	95.1	93.6				105.7	104.0
	J.G.	2	1	92.6	87.5				102.9	97.2
	C.H.	2	1	91.8	91.2				102.0	101.3
	J.G.	3	1	95.5	88.5				106.1	98.3
	J.G.	1	2	94.7	89.5				105.2	99.4
	C.H.	1	2	86.4	91.9				96.0	102.1
	Y.W.	2	2	92.4	97.4				102.7	108.2
	C.H.	2	2	88.4	89.6				98.2	99.6
	Y.W.	3	2	96.9	98.8				107.7	109.8
	C.H.	3	2	91.2	89.9				101.3	99.9
**Mean**						**90.2**	**4.02**	**4.46**	**100.3**	

Supplementary Table S3: Results of individual measurements for the evaluation of intermediate precision

API	Conc. [%]	Result [%]						Mean recovery (n=3) [%]
		TLC plate 1	TLC plate 2	TLC plate 3	Mean (n=3)	SD (n=3)	RSD (n=3)	
**Atenolol**	50	49.9	48.9	51.7	50.2	1.42	2.83	100.3
	70	68.4	65.1	64.5	66.0	2.10	3.18	94.3
	90	90.2	92.9	86.6	89.9	3.16	3.52	99.9
	100	101.7	102.6	100.5	101.6	1.05	1.04	101.6
	120	119.9	118.2	121.7	119.9	1.75	1.46	99.9
**Ceftriaxone**	50	50.3	49.6	51.5	50.5	0.96	1.90	100.9
	70	72.0	65.6	65.7	67.8	3.67	5.41	96.8
	90	89.3	91.1	87.0	89.1	2.06	2.31	99.0
	100	95.4	98.8	99.5	97.9	2.19	2.24	97.9
	120	120.4	119.3	121.5	120.4	1.10	0.91	100.3
**Cefuroxime axetil**	50	49.3	49.0	49.3	49.2	0.17	0.35	98.4
	70	73.0	72.7	68.3	71.3	2.63	3.69	101.9
	90	91.8	92.6	91.9	92.1	0.44	0.47	102.3
	100	98.7	100.7	96.5	98.6	2.10	2.13	98.6
	120	118.9	118.4	118.9	118.7	0.29	0.24	98.9
**Chloroquine**	50	48.7	48.6	48.7	48.7	0.06	0.12	97.3
	70	71.1	67.4	74.9	71.1	3.75	5.27	101.6
	90	93.7	94.1	93.9	93.9	0.20	0.21	104.3
	100	100.5	99.4	99.2	99.7	0.70	0.70	99.7
	120	117.6	117.3	117.4	117.4	0.15	0.13	97.9
**Ciprofloxacin**	50	49.5	49.4	49.6	49.5	0.10	0.20	99.0
	70	74.2	73.5	72.1	73.3	1.07	1.46	104.7
	90	91.3	91.6	91.1	91.3	0.25	0.28	101.5
	100	95.7	95.2	95.2	95.4	0.29	0.30	95.4
	120	119.2	119.0	119.4	119.2	0.20	0.17	99.3
**Dexamethasone**	50	49.0	49.9	50.6	49.8	0.80	1.61	99.7
	70	69.7	66.7	72.5	69.6	2.90	4.17	99.5
	90	92.7	90.2	88.6	90.5	2.07	2.28	100.6
	100	99.3	98.2	95.9	97.8	1.73	1.77	97.8
	120	118.3	119.9	120.8	119.7	1.27	1.06	99.7
**Fluconazole**	50	49.1	50.3	49.8	49.7	0.60	1.21	99.5
	70	71.4	65.3	69.6	68.8	3.13	4.56	98.2
	90	92.2	89.3	90.6	90.7	1.45	1.60	100.8
	100	100.4	98.0	105.1	101.2	3.61	3.57	101.2
	120	118.6	120.4	119.7	119.6	0.91	0.76	99.6
**Furosemide**	50	50.3	51.3	48.8	50.1	1.26	2.51	100.3
	70	74.7	70.0	67.9	70.9	3.48	4.91	101.2
	90	89.3	87.4	93.4	90.0	3.07	3.41	100.0
	100	98.3	98.4	102.6	99.8	2.45	2.46	99.8
	120	120.4	121.4	117.8	119.9	1.86	1.55	99.9
**Glibenclamide**	50	49.9	49.1	48.7	49.2	0.61	1.24	98.5
	70	70.1	72.6	68.5	70.4	2.07	2.94	100.6
	90	90.2	92.4	93.7	92.1	1.77	1.92	102.3
	100	99.0	98.9	99.6	99.2	0.38	0.38	99.2
	120	119.9	118.5	117.6	118.7	1.16	0.98	98.9
**Hydrochlorothiazide**	50	49.1	49.8	49.0	49.3	0.44	0.88	98.6
	70	68.9	74.1	72.8	71.9	2.71	3.76	102.8
	90	92.3	90.5	92.6	91.8	1.14	1.24	102.0
	100	103.0	106.5	102.4	104.0	2.21	2.13	104.0
	120	118.6	119.7	118.4	118.9	0.70	0.59	99.1
**Metformin**	50	49.0	51.6	49.7	50.1	1.35	2.69	100.2
	70	75.6	73.3	76.3	75.1	1.57	2.09	107.2
	90	92.6	86.8	90.8	90.1	2.97	3.30	100.1
	100	101.5	97.3	100.4	99.7	2.18	2.18	99.7
	120	118.4	121.6	119.5	119.8	1.63	1.36	99.9
**Metronidazole**	50	51.1	49.9	50.2	50.4	0.62	1.24	100.8
	70	72.0	69.2	70.9	70.7	1.41	2.00	101.0
	90	87.8	90.2	89.6	89.2	1.25	1.40	99.1
	100	101.3	99.4	101.0	100.6	1.02	1.02	100.6
	120	121.2	119.9	120.2	120.4	0.68	0.57	100.4
**Sulfamethoxazole**	50	49.4	49.9	48.7	49.3	0.60	1.22	98.7
	70	71.4	70.5	73.0	71.6	1.27	1.77	102.3
	90	91.6	90.2	93.6	91.8	1.71	1.86	102.0
	100	107.3	92.3	99.7	99.8	7.50	7.52	99.8
	120	119.0	119.9	117.6	118.8	1.16	0.98	99.0
**Trimethoprim**	50	49.3	51.8	49.1	50.1	1.50	3.00	100.1
	70	72.4	71.5	69.9	71.3	1.27	1.78	101.8
	90	91.7	86.4	92.4	90.2	3.28	3.64	100.2
	100	105.0	96.1	100.5	100.5	4.45	4.43	100.5
	120	119.0	121.8	118.5	119.8	1.78	1.49	99.8
**Mean**							**1.99**	**100.1**

Supplementary Table S4: Results of individual measurements for the evaluation of linearity

API	Modification	Result [%]								Difference to standard condition [%]
		TLC plate 1		TLC plate 2		TLC plate 3		Mean	RSD	
		left spot	right spot	left spot	right spot	left spot	right spot			
Chloroquine	Standard conditions	94.3	91.3	95.5	89.5	92.6	89.6	92.1	2.67	
	Cropping: full plate (with labelling)	91.0	93.7	93.9	95.5	92.7	95.4	93.7	1.81	1.6
	Cropping: without labelling	91.6	90.4	90.6	87.7	97.7	91.1	91.5	3.63	-0.6
	Manual spot detection	96.1	92.4	94.4	91.7	95.0	92.6	93.7	1.84	1.6
	UV lamp not central	92.4	92.0	94.1	89.7	99.2	86.9	92.4	4.51	0.2
	Low battery charge of UV lamp	88.3	89.6	89.5	90.1	88.0	90.4	89.3	1.08	-2.8
	Different box (from Pidinger Werkstätten)	91.2	89.1	89.7	87.9	91.8	92.6	90.4	1.97	-1.8
	Different smartphone for photo and analysis (Fairphone 3)	86.6	88.4	90.4	84.8	91.7	87.0	88.2	2.90	-4.0
Dexametha-sone	Standard conditions	92.2	88.4	90.0	87.1	89.2	87.4	89.1	2.12	
	Cropping: full plate (with labelling)	96.2	88.8	93.4	88.3	93.5	91.3	91.9	3.31	2.9
	Cropping: without labelling	91.9	87.5	90.9	86.5	89.3	87.4	88.9	2.42	-0.1
	Manual spot detection	92.4	88.2	94.2	88.9	96.7	91.0	91.9	3.51	2.9
	UV lamp not central	93.3	90.7	98.9	90.8	97.2	88.4	93.2	4.39	4.2
	Low battery charge of UV lamp	84.3	88.8	88.6	90.3	87.1	86.9	87.7	2.36	-1.4
	Different box (from Pidinger Werkstätten)	88.3	90.8	90.5	90.6	89.4	88.3	89.7	1.29	0.6
	Different smartphone for photo and analysis (Fairphone 3)	92.8	91.0	87.7	90.5	89.1	85.6	89.5	2.86	0.4
Hydrochloro-thiazide	Standard conditions	91.4	93.4	94.5	93.5	93	92.5	93.1	1.12	
	Cropping: full plate (with labelling)	90.7	93.2	96.4	93.1	94.4	91.9	93.3	2.12	0.2
	Cropping: without labelling	88.8	91.8	92.3	93.7	92.8	91.4	91.8	1.82	-1.3
	Manual spot detection	94.8	89.6	95.3	89.3	96.6	90.0	92.6	3.57	-0.4
	UV lamp not central	90.9	86.9	99	91.9	98.1	90.5	92.9	5.07	-0.2
	Low battery charge of UV lamp	94.0	84.1	90.7	82.0	89.2	90.7	88.5	5.11	-4.6
	Different box (from Pidinger Werkstätten)	91.6	90.5	91.3	90.5	92.8	90.2	91.2	1.06	-1.9
	Different smartphone for photo and analysis (Fairphone 3)	92.2	90.4	85.3	89.2	87.8	91.3	89.4	2.82	-3.7
Metformin	Standard conditions	91.7	86.6	88.6	87.9	92.9	88.0	89.3	2.75	
	Cropping: full plate (with labelling)	91.6	88.2	91.5	90.4	91.4	91.2	90.7	1.44	1.4
	Cropping: without labelling	88.2	85.9	90.7	88.9	91.5	88.8	89.0	2.22	-0.3
	Manual spot detection	90.8	88.4	90.8	88.1	91.7	89	89.8	1.66	0.5
	UV lamp not central	89.4	89.1	89.5	87.6	92.7	88.1	89.4	2.00	0.1
	Low battery charge of UV lamp	96.5	88.9	92.7	88.1	91.3	88.1	90.9	3.63	1.7
	Different box (from Pidinger Werkstätten)	91.1	89.3	90.0	90.5	88.1	89.6	89.8	1.16	0.5
	Different smartphone for photo and analysis (Fairphone 3)	91.1	87.9	95.4	89.1	89.1	89.8	90.4	2.95	1.1
**Mean**								**90.8**	**2.60**	

Supplementary Table S5: Results of individual measurements for the evaluation of robustness

API; content by HPLC [% of declared amount]	Brand name; stated manufacturer	No. of photo	Result [%]						Mean (n=12)	Mean Reco-very [%]	RSD [%]
			TLC plate 1		TLC plate 2		TLC plate 3				
			left spot	right spot	left spot	right spot	left spot	right spot			
Finished pharmaceutical products from the pharmacy of the Tübingen University hospital:											
**Dexamethasone; 98.9%**	Dexamethason TAD 4mg; TAD Pharma GmbH	1	99.3	100.0	97.7	101.2	98.5	93.3	98.8	99.9	3.01
		2	101.9	95.4	103.0	97.1	102.2	96.5			
**Hydrochloro-thiazide; 93.3%**	HCT AAA 12,5; AAA Pharma GmbH	1	92.1	92.8	95.4	89.6	99.2	95.1	93.3	100.0	3.10
		2	92.8	94.3	91.1	88.3	94.9	93.8			
**Ciprofloxacin; 98.7%**	Ciprofloxacin STADA 250mg; STADA	1	99.6	94.7	103.2	99.4	92.5	96.0	97.4	98.7	3.87
		2	104.5	96.7	97.5	95.0	97.0	92.5			
**Metronidazole; 101.1%**	Metronidazol STADA 400mg; STADA	1	101.0	95.1	101.4	103.8	104.1	99.6	100.6	99.5	3.52
		2	103.1	96.9	105.1	102.8	94.5	99.6			
									**Mean**	**99.5**	**3.38**
Substandard pharmaceutical products collected in the DR Congo:											
**Ciprofloxacin; 83.4%**	Ciprox 500 Zenufa Laboratoire Batch no. 16T-141	1	87.6	87.9	80.2	83.0	86.9	79.4	84.2	101.0	3.44
		2	87.2	83.5	84.9	81.2	84.3	84.3			
**Metronidazole; 86.7%**	Metrosim-200; Strides Shasun Limited Batch no. 7351898	1	82.6	87.7	83.3	88.8	80.8	83.4	86.0	99.2	4.59
		2	86.8	85.2	81.3	88.9	94.0	89.5			
									**Mean**	**100.1**	**4.01**

Supplementary Table S6: Results of TLCyzer analysis of four finished pharmaceutical products of good quality, and of two substandard pharmaceutical products. Onto each TLC plate, three reference solutions containing 60, 80 and 100% of the standard concentrations given in Table 1, and two sample solutions were applied (see Methods).

API; content by HPLC [% of declared amount]	Brand name; stated manufacturer	No. of photo	Result [%]						Mean (n=12)	Mean Reco-very [%]	RSD [%]
			TLC plate 1		TLC plate 2		TLC plate 3				
			left spot	right spot	left spot	right spot	left spot	right spot			
Finished pharmaceutical products from the pharmacy of the Tübingen University hospital:											
**Dexamethasone; 98.9%**	Dexamethason TAD 4mg; TAD Pharma GmbH	1	92.2	94.8	100.9	98.4	99.8	91.4	95.8	96.8	4.28
		2	90.7	93.3	97.6	98.4	101.3	90.3			
**Hydrochloro-thiazide; 93.3%**	HCT AAA 12,5; AAA Pharma GmbH	1	99.4	97.8	94.3	95.9	94.7	95.0	95.9	102.8	2.67
		2	100.5	96.4	97.0	92.1	95.7	92.1			
**Ciprofloxacin; 98.7%**	Ciprofloxacin STADA 250mg; STADA	1	105.3	102.0	100.8	92.8	102.5	103.1	101.3	102.7	3.94
		2	101.7	97.9	103.3	95.9	104.1	106.7			
**Metronidazole; 101.1%**	Metronidazol STADA 400mg; STADA	1	91.5	93.6	96.6	95.0	95.2	96.1	94.2	93.2	3.49
		2	94.7	90.3	91.6	91.2	92.3	102.3			
									**Mean**	**98.9**	**3.60**
Substandard pharmaceutical products collected in the DR Congo:											
**Ciprofloxacin; 83.4%**	Ciprox 500 Zenufa Laboratoire	1	87.1	88.3	83.8	79.1	84.5	83.5	84.1	100.8	2.98
		2	85.9	82.7	85.2	82.6	84.6	81.3			
**Metronidazole; 86.7%**	Metrosim-200; Strides Shasun Limited	1	85.5	83.6	90.3	83.3	83.7	84.8	84.2	97.1	3.08
		2	83.6	81.9	85.3	80.5	81.5	86.5			
									**Mean**	**99.0**	**3.03**

Supplementary Table S7: Results of TLCyzer analysis using 80% and 100% reference solutions only. The same products as shown in Supplementary Table S6 were examined. For this experiment, onto each TLC plate two reference solutions containing 80 and 100% of the standard concentrations given in Table 1, and two sample solutions were applied (see Methods).
