# Supplementary figures and images for: An open-source smartphone app for the quantitative evaluation of thin-layer chromatographic analyses in medicine quality screening
Source: Sci Rep. 2022 Aug 4;12:13433. doi: 10.1038/s41598-022-17527-y (PMC9352711; doi:10.1038/s41598-022-17527-y)

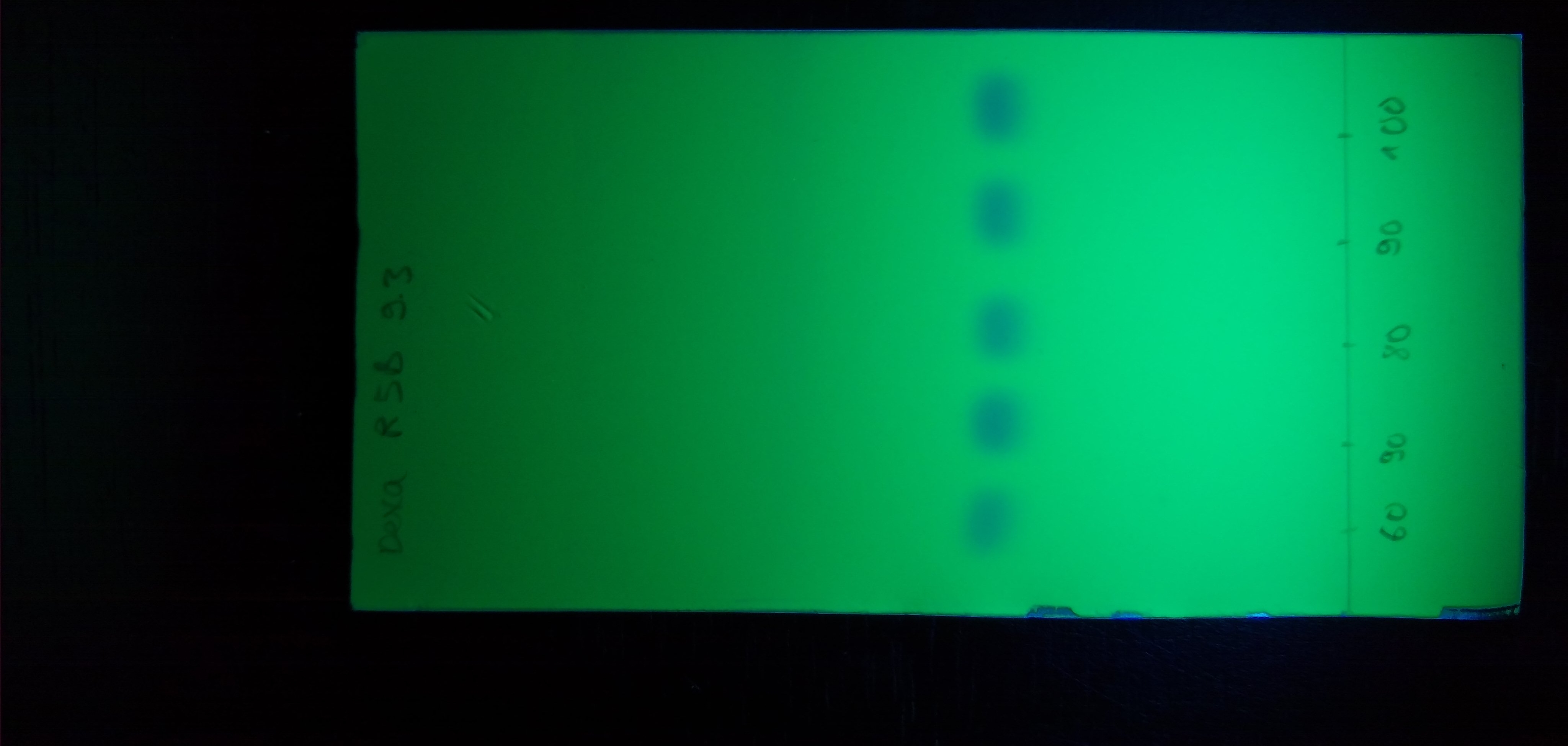

Supplement: Supplementary file 2 — Supplementary TLC Photo Dexamethasone. [file 41598_2022_17527_MOESM2_ESM.jpg]

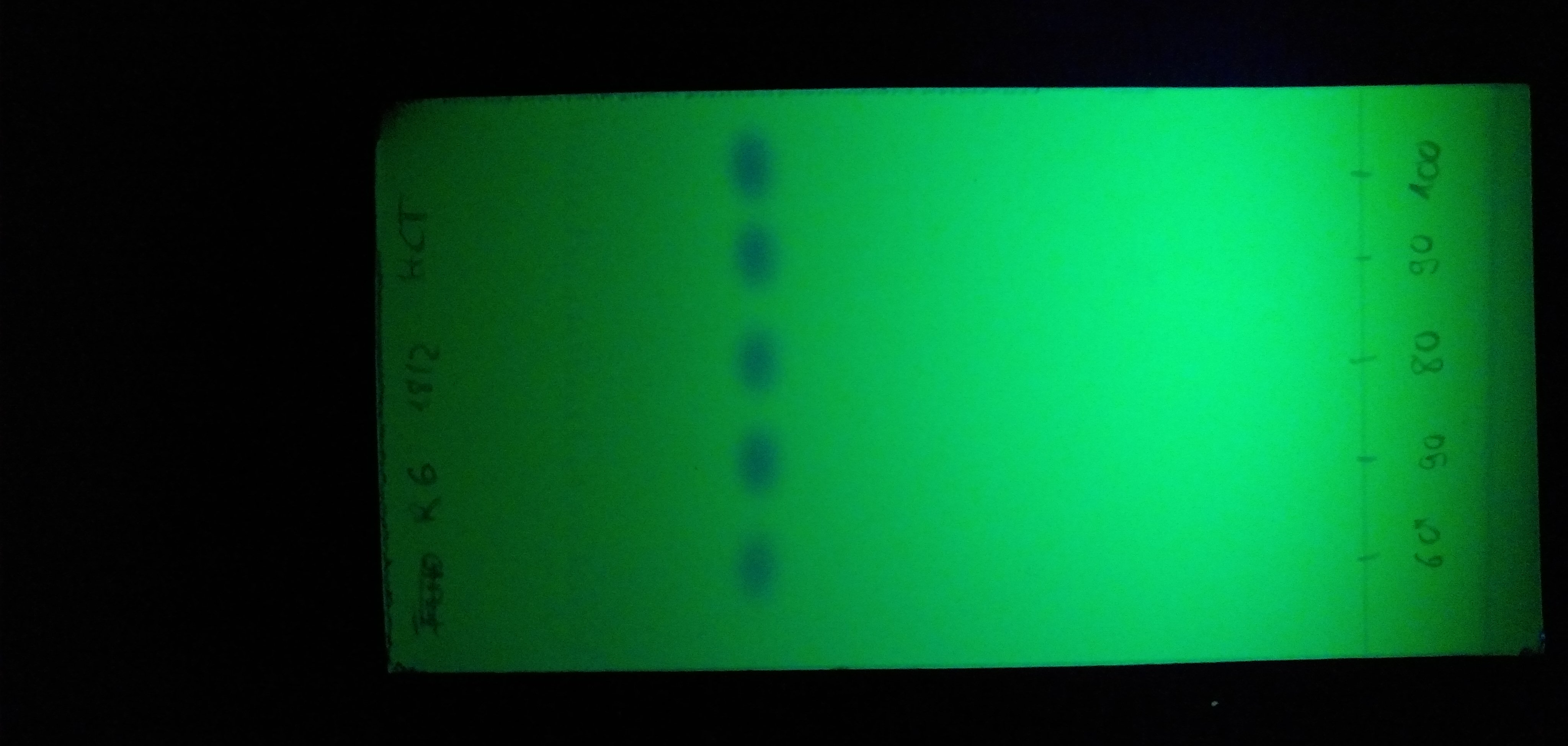

Supplement: Supplementary file 3 — Supplementary TLC Photo Hydrochlorothiazide. [file 41598_2022_17527_MOESM3_ESM.jpg]

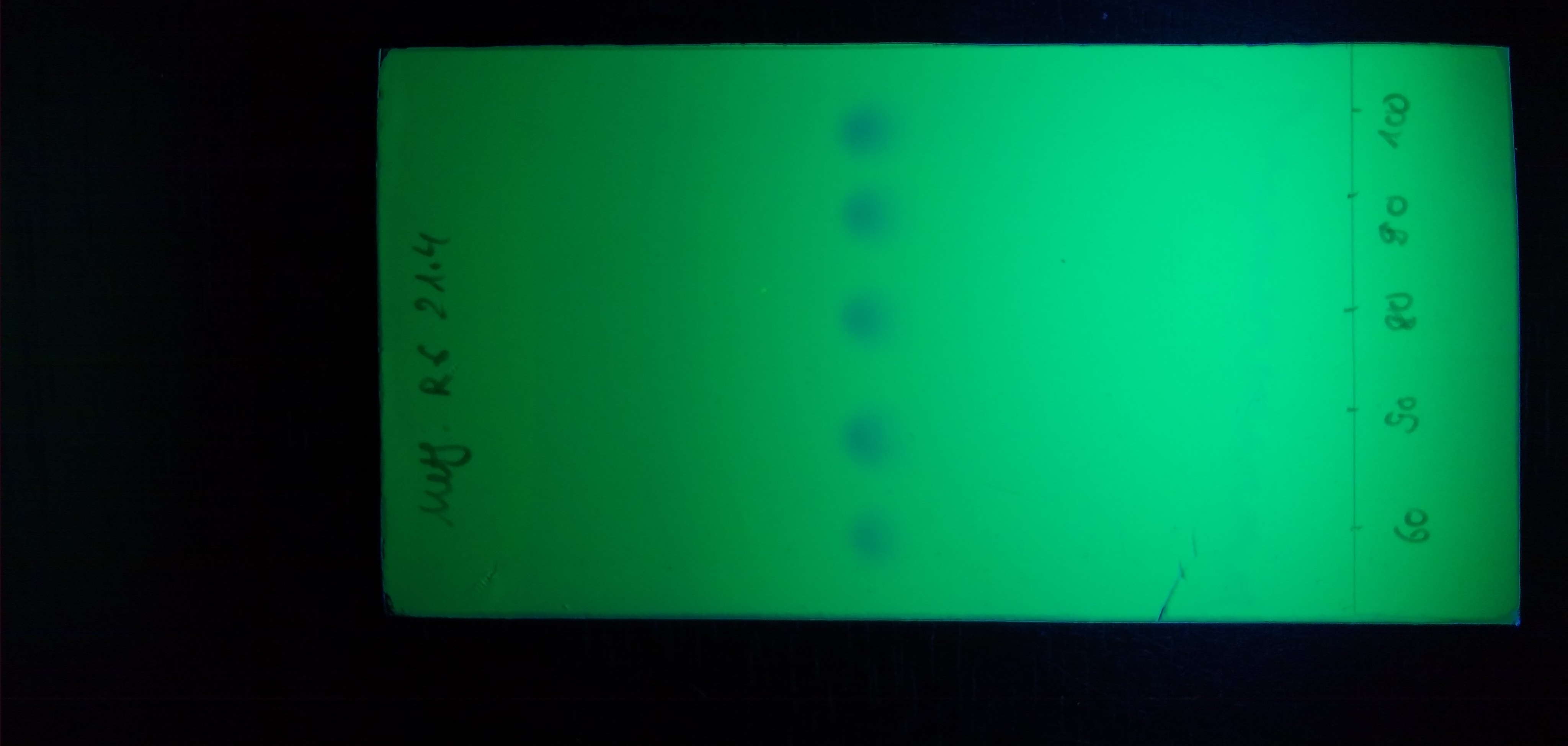

Supplement: Supplementary file 4 — Supplementary TLC Photo Metformin. [file 41598_2022_17527_MOESM4_ESM.jpg]
